# Falkner–Skan Flow with Stream-Wise Pressure Gradient and Transfer of Mass over a Dynamic Wall

**DOI:** 10.3390/e23111448

**Published:** 2021-10-31

**Authors:** Muhammad Fawad Khan, Muhammad Sulaiman, Carlos Andrés Tavera Romero, Ali Alkhathlan

**Affiliations:** 1 Department of Mathematics, Abdul Wali Khan University, Mardan 23200, Pakistan; fawadaurang@gmail.com; 2 COMBA R&D Laboratory, Faculty of Engineering, Universidad Santiago de Cali, Cali 76001, Colombia; carlos.tavera00@usc.edu.co; 3 Computer Science Department, Faculty of Computing and Information Technology, King Abdulaziz University, Jeddah 21589, Saudi Arabia; analkhathlan@kau.edu.sa

**Keywords:** fluid dynamics, numerical methods, computational science, computational fluid dynamics, differential equations, Falkner–Skan system, artificial neural networks, Sine-Cosine Algorithm, sequential quadratic programming, hybrid computing, mass transfer

## Abstract

In this work, an important model in fluid dynamics is analyzed by a new hybrid neurocomputing algorithm. We have considered the Falkner–Skan (FS) with the stream-wise pressure gradient transfer of mass over a dynamic wall. To analyze the boundary flow of the FS model, we have utilized the global search characteristic of a recently developed heuristic, the Sine Cosine Algorithm (SCA), and the local search characteristic of Sequential Quadratic Programming (SQP). Artificial neural network (ANN) architecture is utilized to construct a series solution of the mathematical model. We have called our technique the ANN-SCA-SQP algorithm. The dynamic of the FS system is observed by varying stream-wise pressure gradient mass transfer and dynamic wall. To validate the effectiveness of ANN-SCA-SQP algorithm, our solutions are compared with state-of-the-art reference solutions. We have repeated a hundred experiments to establish the robustness of our approach. Our experimental outcome validates the superiority of the ANN-SCA-SQP algorithm.

## 1. Introduction

Fluid dynamics applies to a large number of fields such as traffic engineering, weather prediction, aerospace, and crowed dynamics [1,2,3,4]. Fluid dynamics can also apply to more complex scenarios, such as in astrophysical problems, including plasma and solar physics. In [5], J. J. González-Avilés et al. present a study about ideal MHD code to study the solar atmosphere and Jet formation in solar atmosphere due to magnetic reconnection [6]. The fluid dynamic behavior depends on the information of velocity, density, temperature, and pressure in terms of space and time. The role of a mathematician is vital to clear the blurred image of fluid dynamics by describing the application of science-based fluid dynamics through mathematical modeling. The Falkner–Skan boundary layer system (FSS) is considered a basic model with many applications in fluid dynamics [7,8,9,10]. The Falkner–Skan system was first presented in 1931 for describing viscous fluid submerged in the flow in overabundance of the stationary wall [11,12]. Generally, the third order differential equation was derived from partial differential equations (PDEs) by performing similarity transformation and analyzing the equations to describe system dynamics [13,14,15,16,17]. Due to the significance of the FS system, numerous analytical and numerical methods are developed for the solution of the FS system. An overview is as follows: The Falkner–Skan system (FSS) was first introduced in 1931 for solution of boundary layer equations [11]. Initially, fewer solutions are available in literature for FSS. The first-ever physical solution for FSS was proposed in 1937 [18]. The irregularities were then reported in 1953 [19]. In 1966, Hertree presented an effective solution of the FS-system [20]. In 1970, another such type of boundary value problem was reported [21]. Moreover, the existence theorem for the solution of the FS system and approximate solution by implementing shooting method was reported in 1971 [22,23]. A random-vortex based method was reported in 1989 [24] and a coordinate transformation reduce input domain with finite difference was proposed in 1998 [25]. In 1999, transformed Navier–Stokes procedure for studying the flow based on FSS is used [26]. Moreover, for reliability of analytical and numerical methods, the FSS was used as a benchmark model. A large number of deterministic methods were reported such as the Fourier series approach [27], homotopy analysis procedure [28], Sinc-collocation methodology [29], and Chebyshev collocation method [30]. Recently, in many problems of fluid dynamics, magneto-hydrodynamics (MHD), nano-fluid and dynamics of Casson fluid FSS arise [31,32,33,34] including SWCNT and MWCNT nano-fluid flow [35], simulation of bioconvection Falkner–Skan flow [36], and asymptotic approximant for the Falkner–Skan [37]. The Falkner–Skan system is studied in different aspects. The presented work analyzed dynamic characteristics of FSS.

In this study, the dynamic characteristics are analyzed in different conditions of streamwise pressure gradient (α), mass wall transfer (μ), and wall movement (δ). The α is the numerical parameter set rate of acceleration or deceleration of main stream, μ is mass transfer over dynamic wall, and δ is wall movement condition. The study is conducted with the following conditions:

- Flow along impermeable wall with zero mass transfer for different accelerated values of main stream.
- Flow for different rate of mass transfer over stationary wall with fixed acceleration of main stream.
- Flow along dynamic wall with zero mass transfer and maximum acceleration of main stream.

In numerical solvers, the stochastic solvers are more efficient and attractive to be implemented as an alternative choice due to the robustness, simplicity of the concept, reliability and easy operation for nonlinear systems based on integer and fractional-order differential equations [38,39,40,41,42,43,44,45,46,47,48,49,50,51]. Numerical stochastic approaches based on soft computing techniques became valuable because of consistency in convergence and accuracy. For an effective optimization process, the role of soft computing based solvers is vital, like binarization methods [52], enhancing re-active power-management [53], fuzzy-controlled-servo systems [54] and optimization procedure of logistic infrastructure based on a mathematical model [55]. The solution of differential equations on the basis of neural network was introduced in 1990. A finite difference method based on neural network was reported in 1990 [56]. Frenandez in 1994 [57,58] presents pioneer work of feed-forward neural network based solution of differential equations. The extension of neural network based solution to PDEs was made in 1998 [59]. In such a way, two types of stochastic numerical solvers were used as hybrid methods i.e., global search and local search, for finding the unknown with reliable and promising solutions. Such as fuel combustion theory [60], Navier–Stokes [61], fractional control problem [62], magneto-hydrodynamics [63], third grade thin film flow [64], Bagley–Torvik fractional order [65], Neural-Network Solution of Single-Delay Differential equations [66], Hamilton–Jacobi differential equations [67], analysis of multi-phase flow through porous media [68], backward stochastic differential equations for pricing and hedging [69], coating dynamics with Oldroyd 8-constant fluid [70], and finite differences based on the neural network [71]. A few of them were implemented for the solution of FSS to study its characteristics. In this work, due to the significance of FSS in various fluid dynamics fields, the FSS is discussed dynamically based on its parameters stream-wise pressure gradient, and the mass wall transfer expresses the dynamics of the mass transfer at the wall and the parameter of wall movement. The stochastic solvers seem reliable and promising for the field of computational fluid dynamics. Their results are accurate and consistent for practical problems based on differential equations. Therefore, for discussing dynamic characteristics of FSS, the stochastic computational solver is inquired based on soft computing terminology.

The significance of the proposed procedure for the solution of FSS based on the stochastic process is addressed as:

- Provides quality, highly reliable, and effective solutions.
- Generally, computational techniques give solutions based on predefined discrete inputs, while the proposed methodology readily produces random inputs in the given entire span.
- No initial guess is required. The proposed scheme is an unsupervised technique.
- Applicable for complex models where the traditional solvers get stuck in a local optimum. The advent of computational methods increased the use of stochastic computational methods for dealing with complicated mathematical models for which conventional methods fail.

The rest of paper is organized as follows: In Section 2, the governing equation and formulation of FS system is discussed. In Section 3, the designed methodology is described. In Section 4, performance matrices are introduced. Section 5 presents a brief graphical and numerical description of different variants of FSS. In Section 6, the presenting methodology is evaluated based on performance matrices while Section 7 concludes the presenting work.

## 2. Formulation of the Falkner–Skan Boundary Layer System

**Figure 1:**
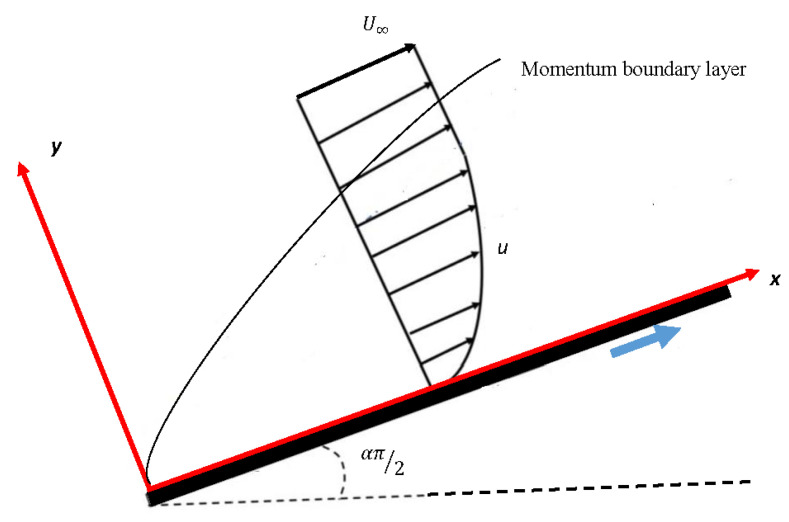
Physical model of FS boundary layer system: The case α=0 becomes the well-known Blasius equation [72], and the case α=1 gives the Hiemenz flow [73].

An incompressible fluid is considered over a wedge, as given in Figure 1. An incompressible fluid referee to a flow in a fluid dynamics in which the density (ρ) is constant within an infinitesimal volume (V) and moves with flow velocity (U(u,v)). In other words, the divergence of velocity is zero, ∇·U=0. The free stream velocity $U_{\infty }$ is uniform and constant. Moreover, the flow is two-dimensional laminar and viscous boundary layer. The continuity equation and boundary layer equations may be written as:

(1) $$\frac{\partial u}{\partial x}+\frac{\partial v}{\partial y}=0,$$

(2) $$u\frac{\partial u}{\partial x}+v\frac{\partial u}{\partial y}=U_{f}\frac{dU_{f}}{dx}+v\frac{\partial^{2}u}{\partial^{2}y}.$$

In Equation (2), *u* is the *x*-component and *v* is the *y*-component of velocity of the fluid flow, and $U_{f}$ is the free stream velocity under pressure gradient at the edge of the boundary layer and is a function of *x*, and the boundary conditions are given by:

(3) $$\begin{array}{cc} \; & \;\mathrm{at}\;y=0:u=v=0,\\ \; & \;\mathrm{at}\;y\to \infty :u\to U_{f}\left(x\right)=U_{\infty }{\left(x/L\right)}^{m},\\ \; & \;\mathrm{at}\;x=0:\;u=U_{\infty }, \end{array}$$

where $U_{\infty }$ is the free mean stream velocity, *L* shows the length of wedge, m represents the Falkner–Skan power law parameter, and *x* is measured from the tip of wedge. For any two-dimensional incompressible flow, the net volume flow rate due to *u* and *v* through a control volume must be zero. In other words, the inlet flow of volume must be equal to outlet flow. Thus, a stream function, Ψ(x,y), is introduced such that

(4) $$\psi_{u}+\psi_{v}=0$$

or    $\psi_{u}=-\psi_{v}\;$ or    $\psi_{v}=-\psi_{u}$,
volume flow rate in the *x*-direction $\to \psi_{u}\to \psi$,
volume flow rate in the *y*-direction $\to -\psi_{u}\to -\psi$,

(5) $$u=\frac{\partial \Psi }{\partial y}\;\;\mathrm{and}\;\;v=-\frac{\partial \Psi }{\partial x}.$$

For the physical considerations which require the introduction of this function, the mathematical significance of its use is that the equation of continuity, i.e., Equation (1), is satisfied identically. The momentum equation becomes:

(6) $$\frac{\partial \Psi }{\partial y}+\frac{\partial^{2}\Psi }{\partial x\partial y}-\frac{\partial \Psi }{\partial x}\frac{\partial^{2}\Psi }{\partial y^{2}}=U_{f}\frac{\partial U_{f}}{\partial x}+v\frac{\partial^{3}\Psi }{\partial y^{3}}.$$

By integrating Equation (5) and introducing a similarity variable yields:

(7) $$g\left(\eta \right)=\sqrt{\frac{1+m}{2}\frac{L^{m}}{vU_{\infty }}}\cdot \left(\Psi /x^{(1+m)/2}\right)$$

(8) $$\eta =\sqrt{\frac{1+m}{2}\frac{U_{\infty }}{vL^{m}}}\cdot \left(y/x^{(1-m)/2}\right).$$

Substituting Equations (7) and (8) into Equation (6) gives the Falkner–Skan boundary layer system. The Falkner–Skan boundary system consists of the Falkner–Skan equation for mass transfer and wall stretching, expressed in terms of a third order nonlinear ordinary differential equation (ODE) as:

(9) $$g^{\prime \prime \prime }+gg^{\prime \prime }+\alpha \left(1-{g^{\prime }}^{2}\right)=0,$$

with boundary conditions

(10) $$g\left(0\right)=\mu ,\;g^{\prime }\left(0\right)=\delta ,\;\mathrm{and}\;g^{\prime }\left(1\right)=1.$$

Here, α is the parameter of streamwise pressure gradient, μ is a parameter of the mass wall transfer, expresses the mass transfer at the dynamic at wall, and δ denotes the parameter of wall movement. g(η) represents the solution of FSS with its first, second, and third derivative $g^{\prime }$, $g^{\prime \prime }$, and $g^{\prime \prime \prime }$, respectively. The variable g is a dimensionless stream function, and the independent variable η is a dimensionless distance from the wall, a so-called similarity variable. Note that, in the equations above, parameters α and *m* are related through the expression α=2m/m+1. The first derivative $g^{\prime }$ defines the dimensionless velocity component in the *x*-direction, the second derivative $g^{\prime \prime }$ defines the dimensionless shear stress in the boundary layer.

## 3. ANN Based Structure of the FS Boundary Layer System

This section consists of the proposed methodology, with a brief description, for the solution of Falkner–Skan boundary value problem based on a stochastic computational method.

**Figure 2:**
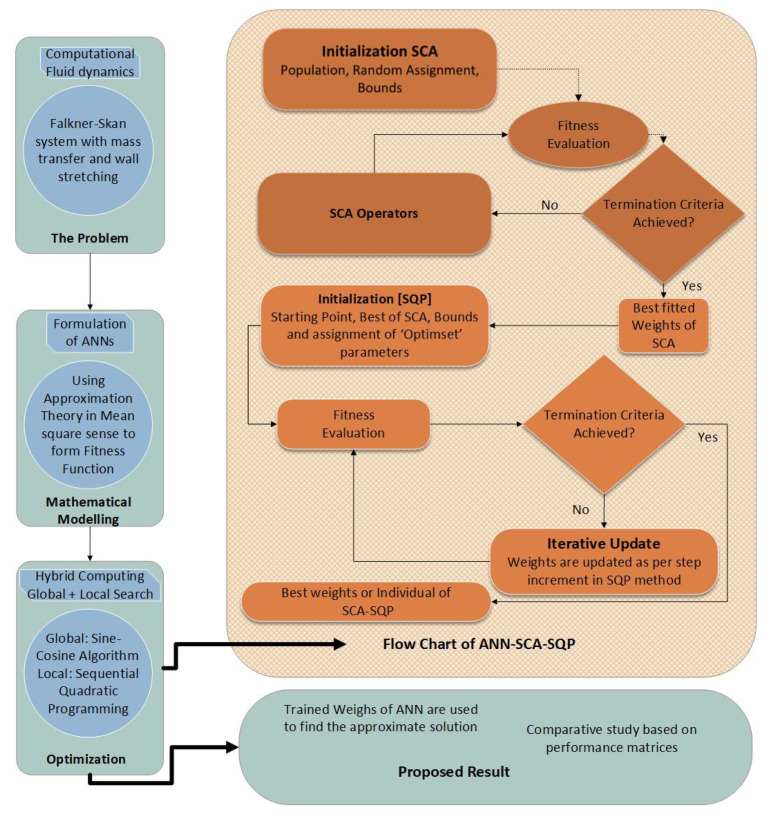
Work flow chart of proposed methodology. Initially, population in SCA is set for generation of solutions, and fitness of the generated solution is evaluated by SCA. The fitted solution is provided as an initial point to SQP, and SQP provides the best solution as weights of ANN.

The designed procedure consists of two phases; the first phase consists of development of the feed-forward Artificial Neural networks (ANN) model in terms of approximation theory for FSS, while the second phase presents the processes of training the weights of ANN. The weights (unknown) are trained with the help of a Sine-Cosine Algorithm (SCA) and Sequential Quadratic Programming (SQP). The work flow chart of presenting methodology is given in Figure 2.

There are two steps of the mathematical model; in the first step, the differential equation Artificial neural networks is designed, while, in the second step, objective/fitness function for the problem is constructed using unsupervised errors.

The structure of the mathematical model for the FS-system is designed by extensively applying the worth of feed-forward ANN. Feed-forward ANN is a less complex, fast unidirectional, and highly responsive to noisy data. The unidirectional process of a feed-forward neural network helps SCA-SQP in convergence. Because SCA generates random solutions, the multi-propagation may affect its performance. ANN is used to solve the FS-system. Its solution g(η) and its derivatives first, second, third, and *n*th order $g^{\prime }\left(\eta \right)$, $g^{\prime \prime }\left(\eta \right)$, $g^{\prime \prime \prime }\left(\eta \right)$ and $g^{n}\left(\eta \right)$, respectively, are given by:

(11) $$\begin{array}{c} \hat{g}\left(\eta \right)=\sum_{i=1}^{k}a_{i}\phi \left(w_{i}\left(\eta \right)+b_{i}\right)\\ \hat{g^{\prime }}\left(\eta \right)=\sum_{i=1}^{k}a_{i}\phi^{\prime }\left(w_{i}\left(\eta \right)+b_{i}\right)\\ \hat{g^{\prime \prime }}\left(\eta \right)=\sum_{i=1}^{k}a_{i}\phi^{\prime \prime }\left(w_{i}\left(\eta \right)+b_{i}\right)\\ .\\ .\\ .\\ \hat{g^{n}}\left(\eta \right)=\sum_{i=1}^{k}a_{i}\phi^{n}\left(w_{i}\left(\eta \right)+b_{i}\right), \end{array}$$

Here, in the model, *k* denotes number of neurons in the network, ϕ denotes activation function and vector W=[a,w,b] represents the unknown (weights) with elements $a=\left[a_{1},a_{2},\ldots ,a_{k}\right],w=\left[w_{1},w_{2},\ldots ,w_{k}\right]$ and $b=[b_{1},b_{2},\ldots ,b_{k}]$. In the neural network procedure, log-sigmoid function is taken as activation function. Mathematically, log-sigmoid function is given as:

(12) $$\phi \left(x\right)=\frac{1}{1+e^{-x}}.$$

As ϕ is taken as an activation function, its derivatives will be also taken as activation functions. The FSS in Equation (9) is based on third order nonlinear ODE, so the log-sigmoid based activation function for the solution of FSS i.e., g(η) and its derivatives i.e., first $g^{\prime }\left(\eta \right)$, second $g^{\prime \prime }\left(\eta \right)$, and third $g^{\prime \prime \prime }\left(\eta \right)$ can be expressed, respectively, as:

(13) $$\hat{g}\left(\eta \right)=\sum_{i=1}^{k}a_{i}\left(\frac{1}{1+e^{-(w_{i}\eta +b_{i})}}\right),$$

(14) $${\hat{g}}^{\prime }\left(\eta \right)=\sum_{i=1}^{k}a_{i}w_{i}\left(\frac{e^{-(w_{i}\eta +b_{i})}}{{\left(1+e^{-(w_{i}\eta +b_{i})}\right)}^{2}}\right),$$

(15) $${\hat{g}}^{\prime \prime }=\sum_{i=1}^{k}a_{i}w_{i}^{2}\left(\frac{2e^{-2\left(w_{i}\eta +b_{i}\right)}}{{\left(1+e^{-\left(w_{i}\eta +b_{i}\right)}\right)}^{3}}-\frac{e^{-\left(w_{i}\eta +b_{i}\right)}}{{\left(1+e^{-\left(w_{i}\eta +b_{i}\right)}\right)}^{2}}\right),$$

(16) $${\hat{g}}^{\prime \prime \prime }=\sum_{i=1}^{k}a_{i}w_{i}^{3}\left(\frac{3!e^{-3\left(w_{i}\eta +b_{i}\right)}}{{\left(1+e^{-\left(w_{i}\eta +b_{i}\right)}\right)}^{4}}-\frac{2!\left(1+2\right)e^{-2\left(w_{i}\eta +b_{i}\right)}}{{\left(1+e^{-\left(w_{i}\eta +b_{i}\right)}\right)}^{3}}+\frac{e^{-\left(w_{i}\eta +b_{i}\right)}}{{\left(1+e^{-\left(w_{i}\eta +b_{i}\right)}\right)}^{2}}\right).$$

**Figure 3:**
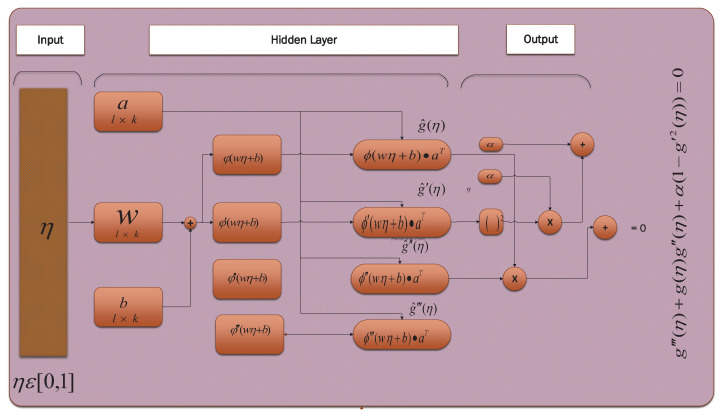
ANN structure with its parameters’ inputs, hidden layer, and outputs. Values of η are inputs. These inputs are transferred to the hidden layer in the form of weights *a*, *w*, and *b* to sigmoid function ϕ, which approximates the solution as output.

Here, Equations (13)–(16) represent arbitrary formulation designed for a neural network of the Falkner–Skan System. The designed structure with its parameters i.e., input, hidden layer, and output, is given in Figure 3.

The fitness function for Falkner–Skan system in terms of two mean-square errors can be expressed as:

(17) $$min\;e=e_{1}+e_{2},$$

where $e_{1}$ is the cost function, can be written as:

(18) $$\begin{array}{c} e_{1}=\frac{1}{N}\sum_{m=1}^{N}{\left({\hat{g}}_{m}^{\prime \prime \prime }+{\hat{g}}_{m}{\hat{g}}_{m}^{\prime }+\alpha \left(1-{\hat{g}}_{m}^{\prime }\right)\right)}^{2},\;\eta \in \left(0,1\right),\\ N=\frac{1}{h},\;{\hat{g}}_{m}=\hat{g}\left(\eta_{m}\right),\;\eta_{m}=\mathrm{mh}, \end{array}$$

where *N* expresses points in the grid depending on step size *h* in given span for inputs, $\hat{g}\left(\eta \right)$, ${\hat{g}}^{\prime }\left(\eta \right)$, ${\hat{g}}^{\prime \prime }\left(\eta \right)$, and ${\hat{g}}^{\prime \prime \prime }\left(\eta \right)$ are shown in Equations (13)–(16). In the same manner, $e_{2}$ is the error function associated with boundary conditions written as:

(19) $$e_{2}=\frac{1}{3}\left({\left({\hat{g}}_{0}-\mu \right)}^{2}+{\left({\hat{g}}_{0}^{\prime }-\delta \right)}^{2}+{\left({\hat{g}}_{N}^{\prime }-1\right)}^{2}\right).$$

**Figure 4:**
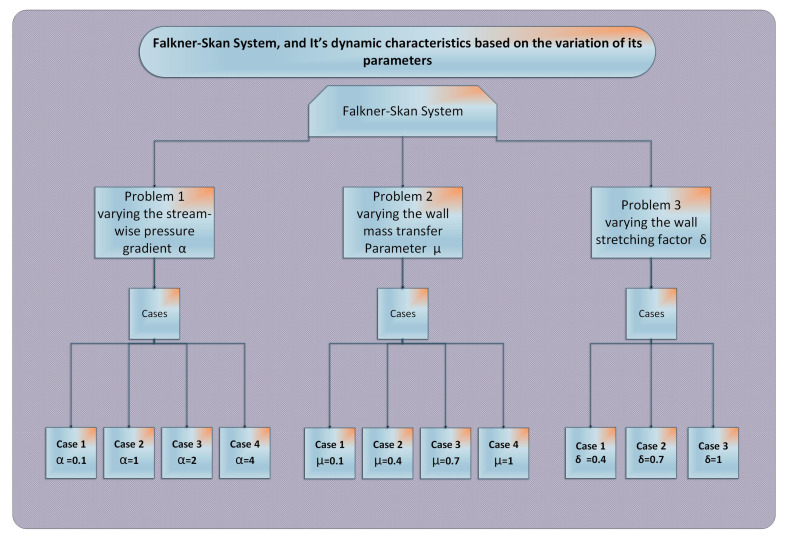
Description of problems of FSS on the basis of variation in parameters. α is a numerical parameter of acceleration or deceleration of the main stream, μ is a condition of mass flow rate, and δ is the parameter of wall movement.

With the provision of such weights, W=[a,w,b], that objective function (*e*) tends to zero, as the two mean square errors $e_{1}$ and $e_{2}$ tend to zero, then the proposed numerical solution $\hat{g}\left(\eta \right)$ tends to the reference solution g(η) of the FSS. Variants of FSS are given in Figure 4 based on its parameters’ streamwise pressure gradient (α), the mass transfer over the dynamic wall (μ), and the parameter of wall dynamics (δ).

### Optimization Procdure

The optimization procedure for a designed structure is performed with the help of a Sine-Cosine Algorithm (SCA) hybrid with local search-method through the Sequential Quadratic Programming (SQP). Due to multi-dimensional capability, the global performance of SCA is better in comparison with other solvers. The solutions of SCA were found reliable. The method was presented by Mirjalili [74]. Few new applications are addressed effectively based on SCA such as a unit commitment problem [75] and crystal wave guides [76], fuzzy probabilistic c-ordered means [77], and for training of multi-layer perceptrons [78]. Enhancing the performance of SCA is hybridized with a local search mechanism, Sequential Quadratic Programming. The Sequential Quadratic Programming solver lays in the category of quadratic optimization solvers implemented for solutions of nonlinear constrained problems. In addition, it is later implemented for many optimization problems such as constrained nonlinear control allocation [79], for the estimation of nonlinear least-squares [80], nonlinear electric circuit models using neural networks based on genetic algorithm and SQP [81], etc., and optimization methodology is described in Figure 2. The pseudo-code of the proposed constructed mechanism for optimization of the objective function, ANN-SCA-SQP algorithm, is written in Figure 1. In this work, two mechanisms, unsupervised and supervised, are hybridized based on the ANN-SCA-SQP algorithm (see Algorithm 1) to find the unknown or weights of the constructed system model for the solution of variants of FSS. The convergence and accuracy of the method also depend on the tuning of parameters; therefore, it is necessary to carefully set the parameters with much experimentation based on optimization knowledge and better understanding.
**Algorithm 1** Pseudo Code of Optimization Algorithm ANN-SCA-SQP. In which, Tolerance a Stopping Criteria**Start:** Sine-Cosine Algorithm(SCA)
**Inputs:** Unknown(weights) W=[a,w,b] Population $P={\left[W_{1},W_{2},\ldots ,W_{m}\right]}^{T}={\left[\left(a_{1},w_{1},b_{1}\right),\left(a_{2},w_{2},b_{2}\right),\ldots ,\left(a_{m},w_{m},b_{m}\right)\right]}^{T}$, for m number of unknown(weights) *W* in *P* and *T* is stand for transpose. Output: Best weights of SCA, i.e., $W_{b}$ **Begin** → Initialization Randomly generation of vector *W* Consist on real values in provided interval Set of *m* weights vectors formulate the preliminary population *P*. / / Stopping-Criteria (SC) Solver-SC → if achieving one of the following: Fitness value $\to 10^{-16}$. Tol-Fun (Function Tolerance) $\to 10^{-20}$ Tol-Con (Constrained Tolerance) $\to 10^{-20}$. //Main-loop of SCA While any of SC parameter satisfy do → Fitness calculation-step Evaluate objective function e as in Equation (9) for the vector *W*. Repeat for m weights *W* of the population P. → Check for SC If SC achieved, then exit from loop else continues. → Parameters of SCA Update the population and repeat from the fitness evaluation End → Storing step Store the best information vector $W_{b}$ and respective fitness value, time, and function evaluated for the current run of the SCA. **End SCA****Start SQP** → Initialization of SQP Initialize SQP method with $W_{b}$ of SCA as an initial weight(point) vector. Set the Stopping criteria SC: Max-Iter (Maximum iterations), i.e., 1000, Tol-Fun as $10^{-24}$ Tol-Con as $10^{-24}$ and Tolerance in optimization variables(weights), i.e., Tol-*X* as $10^{-16}$, While any of SC Value satisfy do → Next step: Calculation of Fitness Evaluate e values using Equation (9) for the weight vector. → Check for SC step If SC achieved, then exit from loop else continues. → Update step Set ’fmincon’ function with technique ’Sequential Quadratic Programming’ Update weight vector for each step through SQP standard procedures. Repeat procedure from fitness calculation step End SCA → Storing step Store the final weight vector along its fitness value, time, generation consumed and function evaluated for the current run of the SCA-SQP method. **End SQP** **Evaluation:** Execute the mechanism of SCA-SQP for multiple independent runs for generation of sufficient data for reliable and
effective evaluation of performance.


## 4. Performance Matrices

The fitness function is constructed using differential equations based on neural networks in terms of mean square-error as in Equation (17). For the minimization of fitness/objective function, an appropriate set of weights is required. The unknown (weights) are the optimization variable. To minimize the objective function of the problem, these variables should be trained in real valued bounds i.e., constraints. To find an appropriate set of weights, both of the mechanisms i.e., global search and local search, were explored with the help of a meta-heuristic procedure based on Sine-Cosine Algorithm and sequential Quadratic Programming. For the reliable and consistent evaluation of the proposed mechanism, other performance indices based on global version are also adapted.

The performance-evaluators’ mean-absolute derivation (MAD), error in Nash–Sutcliffe efficiency (ENSE), and root-mean square error (RMSE) are implemented to approach the work of the presenting scheme. The mathematical formulation of MAD, ENSE, RMSE, and NSE are given as:

(20) $$MAD=\frac{1}{n}\sum_{i=1}^{n}\left|g\left(\eta_{i}\right)-\hat{g}\left(\eta_{i}\right)\right|,$$

(21) $$RMSE=\sqrt{\frac{1}{n}\sum_{i=1}^{n}{\left(g\left(\eta_{i}\right)-\hat{g}\left(\eta_{i}\right)\right)}^{2}},$$

(22) $$NSE=1-\left(\frac{\sum_{i=1}^{n}{\left(g\left(\eta_{i}\right)-\hat{g}\left(\eta_{i}\right)\right)}^{2}}{\sum_{i=1}^{n}{\left(g\left(\eta_{i}\right)-\frac{1}{n}\sum_{i=1}^{n}\left(g\left(\eta_{i}\right)\right)\right)}^{2}}\right),$$

(23) ENSE=|1−NSE|.

Here, *n* denotes the number of input points in a grid, $\hat{g}\left(\eta \right)$ is the proposed, and g(η) is the reference solution. For a reliable and effective system, the value of performance measures based on MAD, ENSE, and RMSE should be zero, while the NSE value approaches 1.

The global extension of the performance measures discussed above are mathematically defined as:

(24) $$GMAD=\frac{1}{R}\sum_{r=1}^{R}\left(\frac{1}{n}\sum_{i=1}^{n}\left(\left|g\left(\eta_{i}\right)-\hat{g}\left(\eta_{i}\right)\right|\right)\right),$$

(25) $$GRMSE=\frac{1}{R}\sum_{r=1}^{R}\left(\sqrt{\frac{1}{n}\sum_{i=1}^{n}{\left(g\left(\eta_{i}\right)-\hat{g}\left(\eta_{i}\right)\right)}^{2}}\right),$$

(26) $$GENSE=\frac{1}{R}\sum_{r=1}^{R}\left(\frac{\sum_{i=1}^{n}{\left(g\left(\eta_{i}\right)-\hat{g}\left(\eta_{i}\right)\right)}^{2}}{\sum_{i=1}^{n}\left(g\left(\eta_{i}\right)-\frac{1}{n}\sum_{i=1}^{n}\left(g\left(\eta_{i}\right)\right)\right)}\right),$$

(27) $$GFIT=\frac{1}{R}\sum_{r=1}^{R}e_{r},$$

where *R* denotes number of runs and $e_{r}$ is fitness value at the *r*th number run of the proposed method. The standard value of all global operators is zero. The global version is based on the average fitness value. Global operators for fitness are GFIT, for MAD, it is GMAD, for RMSE, it is GRMSE, and, for ENSE, it is GENSE.

## 5. Empirical Results and Discussion

In this section, the empirical results for the ANN based designed scheme with ANN-SCA-SQP algorithm are briefly discussed. To analyze the dynamic behavior of the Falkner–Skan system, three problems are presented based on varying parameters of FSS i.e., streamwise pressure gradient α, the parameter of mass transfer μ, and wall movement parameter δ. For comparison, reference solutions of GA-ASM are taken as standard throughout the study. The problems of FSS are described in Figure 4.

### 5.1. Problem 1: Dynamics of FSS Based on the Variation of Stream-Wise Pressure Gradient α

In this problem, the two parameters’ wall mass transfer μ and wall movement δ are kept fixed by taking μ=0 and δ=0, while the variation of streamwise pressure gradient α formed four cases in this problem. The zero value of μ corresponds to zero mass transfer and δ corresponds to flow along the stationary wall. The degree of acceleration or deceleration of the main stream in the Falkner–Skan system is set by a positive or negative value of parameter α. The flows with zero value for this parameter will be considered, i.e., the flows without longitudinal pressure gradient in the main stream. The inputs are taken between 0 and 1, so the simplified form of the system is given as:

(28) $$g^{\prime \prime \prime }+gg^{\prime \prime }+\alpha \left(1-{g\prime }^{2}\right)=0,$$

(29) $$g\left(0\right)=0,\;g^{\prime }\left(0\right)=0,\;\mathrm{and}\;g^{\prime }\left(1\right)=1.$$

**Figure 5:**
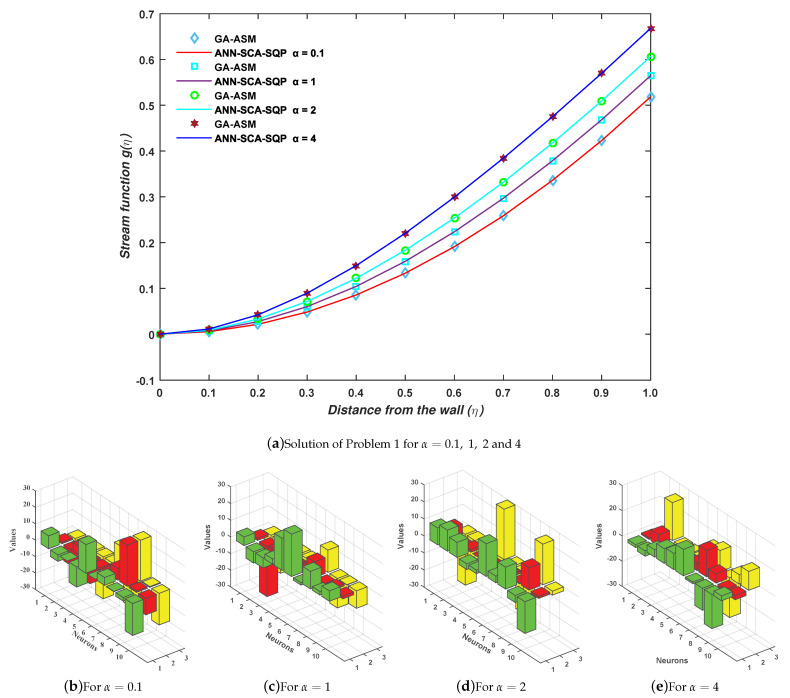
(**a**) Problem 1: Graph between stream function and distance from the wall; (**b**–**e**) the trained unknown (weights) for ANN through the proposed hybrid optimization approach.

The cases are constructed for α=0.1,α=1,α=2andα=4 to analyse the dynamic behavior of FSS in this problem. For each case, the fitness function is designed as per Equation (17). For 11 input points, the fitness function can be written as:

(30) $$e_{c1}=\frac{1}{11}\sum_{m=1}^{11}{\left({\hat{g}}^{\prime \prime \prime }+\hat{g}{\hat{g}}^{\prime \prime }+0.1\left(1-{{\hat{g}}^{\prime }}^{2}\right)\right)}^{2}+\frac{1}{3}\left({\hat{g_{0}}}^{2}+{{\hat{g}}^{\prime }}^{2}+{\left({\hat{g}}_{11}-1\right)}^{2}\right),$$

(31) $$e_{c2}=\frac{1}{11}\sum_{m=1}^{11}{\left({\hat{g}}^{\prime \prime \prime }+\hat{g}{\hat{g}}^{\prime \prime }+\left(1-{{\hat{g}}^{\prime }}^{2}\right)\right)}^{2}+\frac{1}{3}\left({\hat{g_{0}}}^{2}+{{\hat{g}}^{\prime }}^{2}+{\left({\hat{g}}_{11}-1\right)}^{2}\right),$$

(32) $$e_{c3}=\frac{1}{11}\sum_{m=1}^{11}{\left({\hat{g}}^{\prime \prime \prime }+\hat{g}{\hat{g}}^{\prime \prime }+2\left(1-{{\hat{g}}^{\prime }}^{2}\right)\right)}^{2}+\frac{1}{3}\left({\hat{g_{0}}}^{2}+{{\hat{g}}^{\prime }}^{2}+{\left({\hat{g}}_{11}-1\right)}^{2}\right),$$

(33) $$e_{c4}=\frac{1}{11}\sum_{m=1}^{11}{\left({\hat{g}}^{\prime \prime \prime }+\hat{g}{\hat{g}}^{\prime \prime }+4\left(1-{{\hat{g^{\prime }}}^{\prime }}^{2}\right)\right)}^{2}+\frac{1}{3}\left({\hat{g_{0}}}^{2}+{{\hat{g}}^{\prime }}^{2}+{\left({\hat{g}}_{11}-1\right)}^{2}\right),$$

the proposed mechanism, the ANN-SCA-SQP algorithm as discussed in the second section, is implemented for minimization of objective/fitness functions as given in Equations (30)–(33) for four cases $C_{1}$, $C_{2}$, $C_{3}$, and $C_{4}$ of problem 1. For each case, one set of weights is obtained, and putting those sets of weights in Equation (13) gives the solution for each case, as given in Equations (34)–(37) for cases 1, 2, 3, and 4, respectively. The weights are also shown graphically in Figure 5b–e for cases 1, 2, 3, and 4, respectively:

(34) $$g_{c1}\left(\eta \right)=\frac{7.8783}{1+e^{-(0.8860\eta -1.7221)}}+\frac{-3.9499}{1+e^{-(-9.5789\eta -22.8166)}}+\ldots +\frac{-19.4404}{1+e^{-(-9.9739\eta -19.2287)}},$$

(35) $$g_{c2}\left(\eta \right)=\frac{5.1403}{1+e^{-(1.2519\eta -4.7383)}}+\frac{-6.7367}{1+e^{-(-29.9925\eta -19.1042)}}+\ldots +\frac{6.2034}{1+e^{-(-1.6663\eta -10.4633)}},$$

(36) $$g_{c3}\left(\eta \right)=\frac{9.8910}{1+e^{-(6.6099\eta -29.8573)}}+\frac{12.3422}{1+e^{-(0.0286\eta -10.9005)}}+\ldots +\frac{-18.6040}{1+e^{-(-1.1558\eta -2.3439)}},$$

(37) $$g_{c4}\left(\eta \right)=\frac{-2.2571}{1+e^{-(1.1775\eta +0.0490)}}+\frac{-4.1959}{1+e^{-(8.6005\eta -30.0000)}}+\ldots +\frac{3.8141}{1+e^{-(-3.3564\eta +14.9105)}}.$$

**Figure 6:**
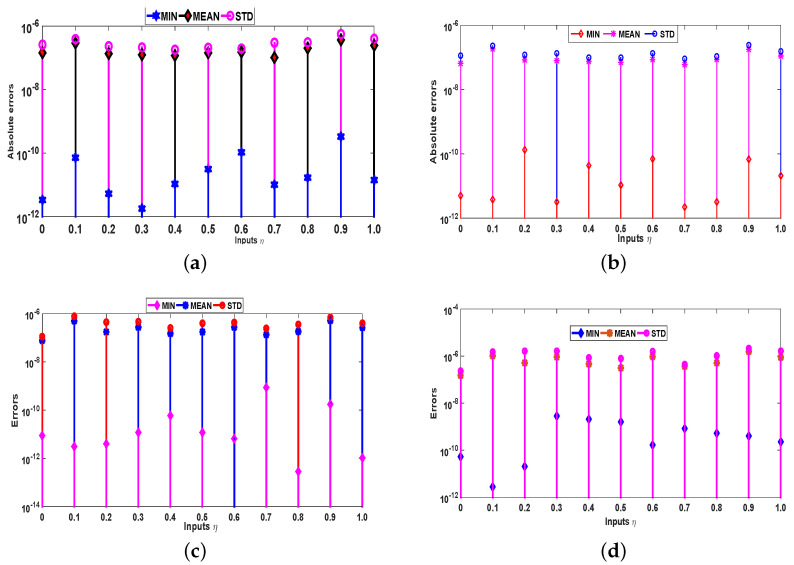
Graphs of statistical data in Table 2. (**a**) For α=0.1; (**b**) For α=1; (**c**) For α=2; (**d**) For α=4.

The full form of Equations (34)–(37) are shown in the Appendix A for up to 14-decimal places. The results of approximate solution in Equations (34)–(37) are graphically shown in Figure 5a for 11 grid points by taking inputs η∈[0,1] having step size 0.1. It is observed that the solutions overlap with the numerical solutions of GA-ASM. The numerical comparison of solutions is also given in Table 1. From solutions, it seems that, with the increase in input η, the stream function g(η) also increases. In other words, we can say that η is directly proportional to g(η):

Moreover, the accuracy and effectiveness of the proposed technique are evaluated by statistical study for 100 independent runs executed by the ANN-SCA-SQP algorithm. In a statistical study based on mean (MEAN), minimum (MIN), and standard deviation (STD), the values of mean and STD seem consistent for each case. For all the cases MIN, mean and STD values are between $10^{-9}$ to $10^{-12}$, $10^{-7}$ to $10^{-8}$, and $10^{-7}$ to $10^{-8}$, respectively, while it is observed that the small decrease in accuracy was found by increasing the value of α. The detailed statistical results are given in Table 2, for graphical illustration of results in Table 2, we have presented Figure 6. The statistical result shows the consistency, reliability, accuracy and convergence.

### 5.2. Problem 2: Dynamics of FSS Based on the Variation of Wall Mass Transfer Parameter μ

In problem 2, the dynamic behavior of FSS, in Equation (9), is evaluated by fixing α=1 and δ=0, while allowing variation in parameter of wall-mass transfer μ. The values of α and δ correspond to accelerated main stream flow along the impermeable wall. The mass-transfer parameter μ in the boundary condition sets the measure for the mass flow rate through the wall boundary in either direction. Positive values determine flows with suction, and negative with blowing through the wall boundary. The FSS is formulated for this case as:

(38) $$g^{\prime \prime \prime }+gg^{\prime }+1-{g^{\prime }}^{2}=0,$$

(39) $$g\left(0\right)=\mu ,\;g^{\prime }\left(0\right)=0,\;\mathrm{and}\;g^{\prime }\left(1\right)=1.$$

The formulation of all cases of the model in Equations (38) and (39) are based on μ=0.1,μ=0.4,μ=0.7andμ=1 and fitness functions for N = 11 are designed as:

(40) $$e_{c1}=\frac{1}{11}\sum_{m=1}^{11}{\left({\hat{g}}_{m}^{\prime \prime \prime }+{\hat{g}}_{m}{\hat{g}}_{m}^{\prime }+\left(1-{\hat{g}}_{m}^{2}\right)\right)}^{2}+\frac{1}{3}\left({\left({\hat{g}}_{0}-0.1\right)}^{2}+{{\hat{g}}^{\prime }}^{2}0+{\left({{\hat{g}}^{\prime }}_{11}^{2}-1\right)}^{2}\right),$$

(41) $$e_{c2}=\frac{1}{11}\sum_{m=1}^{11}{\left({\hat{g}}_{m}^{\prime \prime \prime }+{\hat{g}}_{m}{\hat{g}}_{m}^{\prime }+\left(1-{\hat{g}}_{m}^{2}\right)\right)}^{2}+\frac{1}{3}\left({\left({\hat{g}}_{0}-0.4\right)}^{2}+{{\hat{g}}^{\prime }}^{2}0+{\left({{\hat{g}}^{\prime }}_{11}^{2}-1\right)}^{2}\right),$$

(42) $$e_{c3}=\frac{1}{11}\sum_{m=1}^{11}{\left({\hat{g}}_{m}^{\prime \prime \prime }+{\hat{g}}_{m}{\hat{g}}_{m}^{\prime }+\left(1-{\hat{g}}_{m}^{2}\right)\right)}^{2}+\frac{1}{3}\left({\left({\hat{g}}_{0}-0.7\right)}^{2}+{{\hat{g}}^{\prime }}^{2}0+{\left({{\hat{g}}^{\prime }}_{11}^{2}-1\right)}^{2}\right),$$

(43) $$e_{c4}=\frac{1}{11}\sum_{m=1}^{11}{\left({\hat{g}}_{m}^{\prime \prime \prime }+{\hat{g}}_{m}{\hat{g}}_{m}^{\prime }+\left(1-{\hat{g}}_{m}^{2}\right)\right)}^{2}+\frac{1}{3}\left({\left({\hat{g}}_{0}-1\right)}^{2}+{{\hat{g}}^{\prime }}^{2}0+{\left({{\hat{g}}^{\prime }}_{11}^{2}-1\right)}^{2}\right).$$

**Figure 7:**
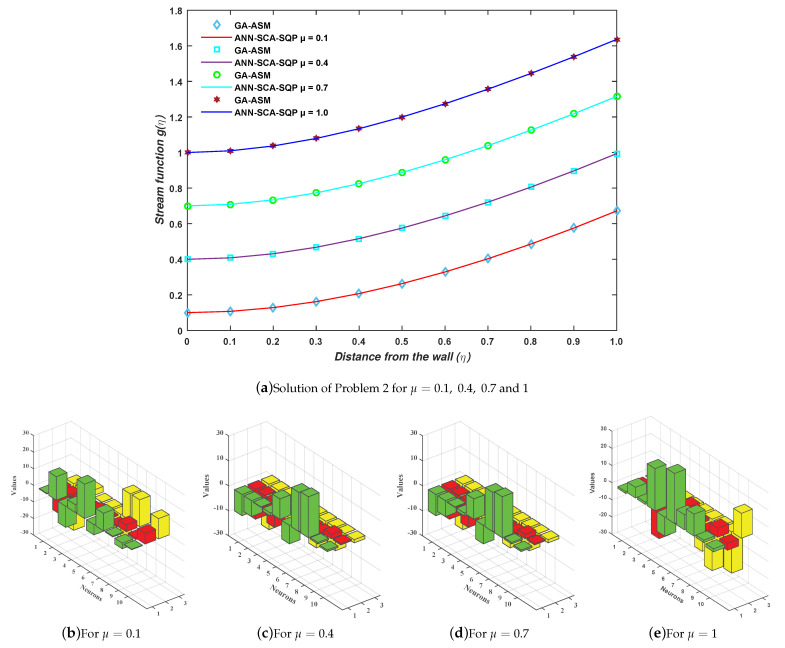
(**a**) Problem 2: Graph between stream function and distance from the wall; (**b**–**e**) the trained unknown (weights) for ANN through proposed hybrid optimization approach.

The proposed mechanism ANN-SCA-SQP algorithm, as discussed in the second section, is implemented for minimization of the fitness function as given in Equations (40)–(43) for four cases $C_{1}$, $C_{2}$, $C_{3}$, and $C_{4}$ of problem 2. For each case, one set of weights is obtained by putting those sets of weights in Equation (13), giving the solution for each case, as given in Equations (44)–(47) for case 1, case 2, case 3, and case 4, respectively. The weights are also shown graphically in Figure 7b–e for cases 1, 2, 3, and 4, respectively:

(44) $$g_{c1}\left(\eta \right)=\frac{0.3931}{1+e^{-(-15.4530\eta -28.1365)}}+\frac{14.1327}{1+e^{-(-1.1618\eta -2.3460)}}+\ldots +\frac{-0.3180}{1+e^{-(6.4054\eta +12.2749)}},$$

(45) $$g_{c2}\left(\eta \right)=\frac{-8.4731}{1+e^{-(-11.9477\eta -18.1566)}}+\frac{-6.3359}{1+e^{-(-0.1630\eta -4.3857)}}+\ldots +\frac{0.5727}{1+e^{-(1.7024\eta +0.0114)}},$$

(46) $$g_{c3}\left(\eta \right)=\frac{-1.0779}{1+e^{-(-1.2854\eta +0.1960)}}+\frac{6.1959}{1+e^{-(-0.14353\eta -10.1054)}}+\ldots +\frac{-1.6986}{1+e^{-(-3.8934\eta -2.8733)}},$$

(47) $$g_{c4}\left(\eta \right)=\frac{-1.2471}{1+e^{-(1.5356\eta +3.5766)}}+\frac{-0.6766}{1+e^{-(0.6557\eta +17.6555)}}+\ldots +\frac{27.6538}{1+e^{-(0.3084\eta -2.2951)}}.$$

The full form of Equations (44)–(47) are written in the Appendix A for up to 14-decimal places. The results of approximate solution in Equations (44)–(47) are graphically shown in Figure 7a for 11 grid points by taking inputs ηϵ[0,1] with step size 0.1. It is observed that the solutions overlap with the numerical solutions of GA-ASM. The numerical comparison of solutions is also given in Table 3.

**Figure 8:**
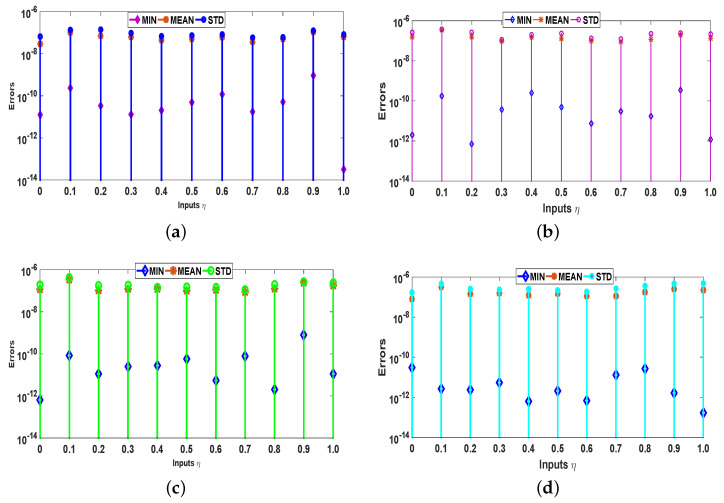
Graphs of statistical data in Table 4. (**a**) For case 1; (**b**) For case 2; (**c**) For case 3; (**d**) For case 4.

Moreover, the reliability and effectiveness of the proposed method are evaluated by statistical study for 100 independent runs executed by the ANN-SCA-SQP algorithm. In a statistical study based on minimum (MIN), mean, and standard deviation (STD), the values of mean and STD seem consistent for each case. For all the cases MIN, mean and STD values are between $10^{-9}$ to $10^{-12}$, $10^{-7}$ to $10^{-8}$, and $10^{-7}$ to $10^{-8}$, respectively, while a small decrease found in accuracy is observed by increasing the value of μ. The detailed statistical results are given in Table 4, for graphical illustration of results in Table 2, we have presented Figure 8. The statistical result shows the consistency, reliability, accuracy and convergence.

### 5.3. Problem 3: Dynamics of FSS Based on the Variation of Wall Stretching Factor δ

In problem 3, the dynamic behavior of FSS in Equation (9) is analyzed by fixing α=1 and μ=0 while allowing variation in wall movement δ parameter. The flow is along the dynamic wall with zero mass transfer and maximum accelerated main stream. The FSS is updated for this problem as:

(48) $$g^{\prime \prime \prime }+gg^{\prime }+1-{g^{\prime }}^{2}=0,$$

(49) $$g\left(0\right)=0,\;g^{\prime }\left(0\right)=\delta ,\;\mathrm{and}\;g^{\prime }\left(1\right)=1.$$

The formulation of cases of the system, Equations (48) and (49), is based on δ=0.4, δ=0.7 and δ=1 and fitness functions for N = 11 are designed as:

(50) $$e_{c1}=\frac{1}{11}\sum_{m=1}^{11}{\left({\hat{g}}_{m}^{\prime \prime \prime }+{\hat{g}}_{m}{\hat{g}}_{m}^{\prime }+\left(1-{\hat{g}}_{m}^{2}\right)\right)}^{2}+\frac{1}{3}\left({\hat{g}}_{0}^{2}+{\left({\hat{g}}_{0}^{\prime }-0.4\right)}^{2}+{\left({{\hat{g}}^{\prime }}_{11}^{2}-1\right)}^{2}\right),$$

(51) $$e_{c2}=\frac{1}{11}\sum_{m=1}^{11}{\left({\hat{g}}_{m}^{\prime \prime \prime }+{\hat{g}}_{m}{\hat{g}}_{m}^{\prime }+\left(1-{\hat{g}}_{m}^{2}\right)\right)}^{2}+\frac{1}{3}\left({\hat{g}}_{0}^{2}+{\left({\hat{g}}_{0}^{\prime }-0.7\right)}^{2}+{\left({{\hat{g}}^{\prime }}_{11}^{2}-1\right)}^{2}\right),$$

(52) $$e_{c3}=\frac{1}{11}\sum_{m=1}^{11}{\left({\hat{g}}_{m}^{\prime \prime \prime }+{\hat{g}}_{m}{\hat{g}}_{m}^{\prime }+\left(1-{\hat{g}}_{m}^{2}\right)\right)}^{2}+\frac{1}{3}\left({\hat{g}}_{0}^{2}+{\left({\hat{g}}_{0}^{\prime }-1\right)}^{2}+{\left({{\hat{g}}^{\prime }}_{11}^{2}-1\right)}^{2}\right).$$

**Figure 9:**
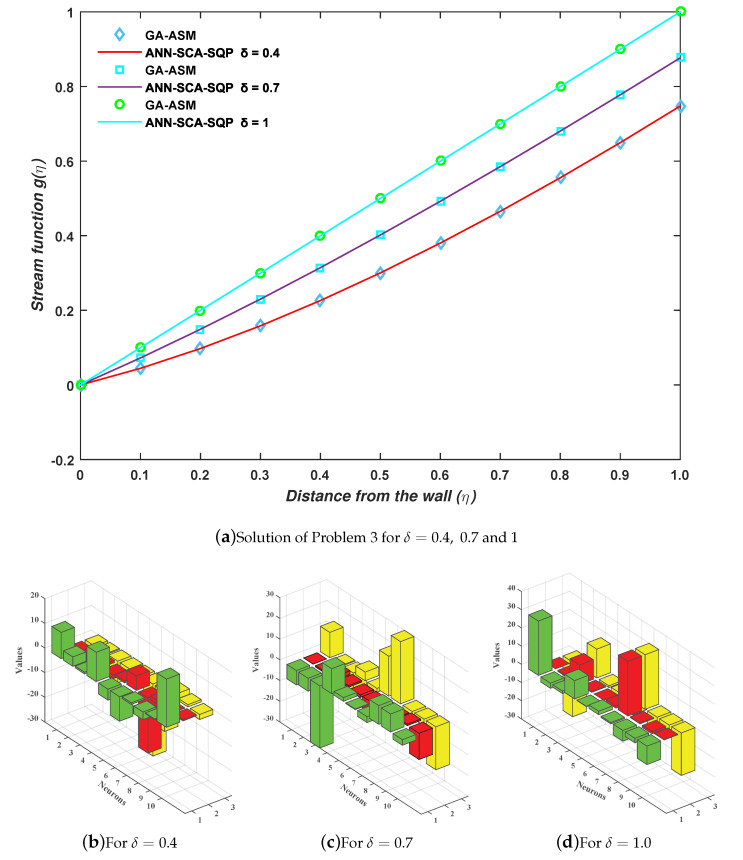
(**a**) Problem 3: Graph between stream function and distance from the wall; (**b**–**d**) the trained unknown (weights) for ANN through the proposed hybrid optimization approach.

The similar procedure is followed for this problem, as for problems 1 and 2, to minimize the fitness functions in Equations (50)–(52). The set of weights is obtained for each case and put in Equation (13). The solutions are shown in Equations (53)–(55). The weights are also shown graphically in Figure 9b–d for cases 1, 2, and 3, respectively.

(53) $$g_{c1}\left(\eta \right)=\frac{10.6223}{1+e^{-(-0.0780\eta -11.0594)}}+\frac{3.7981}{1+e^{-(0.8614\eta -0.4384)}}+\ldots +\frac{19.5012}{1+e^{-(0.4413\eta -2.0520)}},$$

(54) $$g_{c2}\left(\eta \right)=\frac{-6.9289}{1+e^{-(0.0545\eta +12.2082)}}+\frac{-6.2333}{1+e^{-(-0.5813\eta -0.4090)}}+\ldots +\frac{-2.6900}{1+e^{-(-12.6647\eta -20.6995)}},$$

(55) $$g_{c3}\left(\eta \right)=\frac{29.8111}{1+e^{-(-0.0107\eta -30)}}+\frac{-2.7662}{1+e^{-(-0.5871\eta -1.1425)}}+\ldots +\frac{-10.1321}{1+e^{-(-0.2161\eta -23.3399)}}.$$

The full form of Equations (53)–(55) is shown in the Appendix A with up to 14 decimal places. The results of approximate solution in Equations (53)–(55) are graphically shown in Figure 9a for 11 grid points by taking inputs ηϵ[0,1] with step size 0.1. It is observed that the solutions overlap with the numerical solutions of GA-ASM. The numerical comparison of solutions is also given in Table 5.

**Figure 10:**
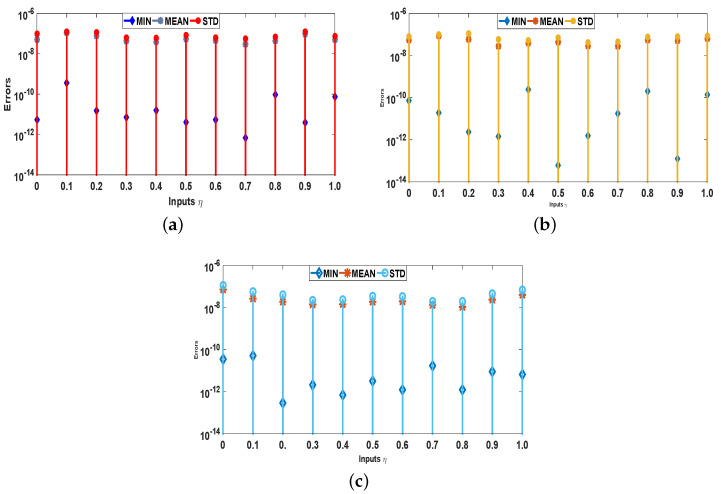
Graphs of statistical data in Table 6. (**a**) For case 1; (**b**) For case 2; (**c**) For case 3.

Moreover, the reliability and effectiveness of the proposed method are evaluated by statistical study for 100 independent runs executed by the ANN-SCA-SQP algorithm. In a statistical study based on minimum (MIN), mean, and standard deviation (STD), the values of mean and STD seem consistent for each case. For all the cases MIN, mean and STD values are between $10^{-10}$ to $10^{-13}$, $10^{-8}$ to $10^{-9}$, and $10^{-7}$ to $10^{-8}$, respectively, while a small decrease found in accuracy is observed by increasing the value of μ. The detailed statistical results are given in Table 6, for graphical illustration of results in Table 6, we have presented Figure 10. The statistical result shows the consistency, reliability, accuracy and convergence.

## 6. Evaluation through Performance Matrices

In this section, for the solution of all three problems, the scheme is executed 100 times and then analyzed comparatively on the basis of performance measures MAD, ENSE, and RMSE along with global extension. The provision of comparative analysis is in terms of convergence accuracy and global performance evaluators. Firstly, the accuracy and convergence are discussed and secondly global performance operators.

### 6.1. Accuracy and Convergence

**Figure 11:**
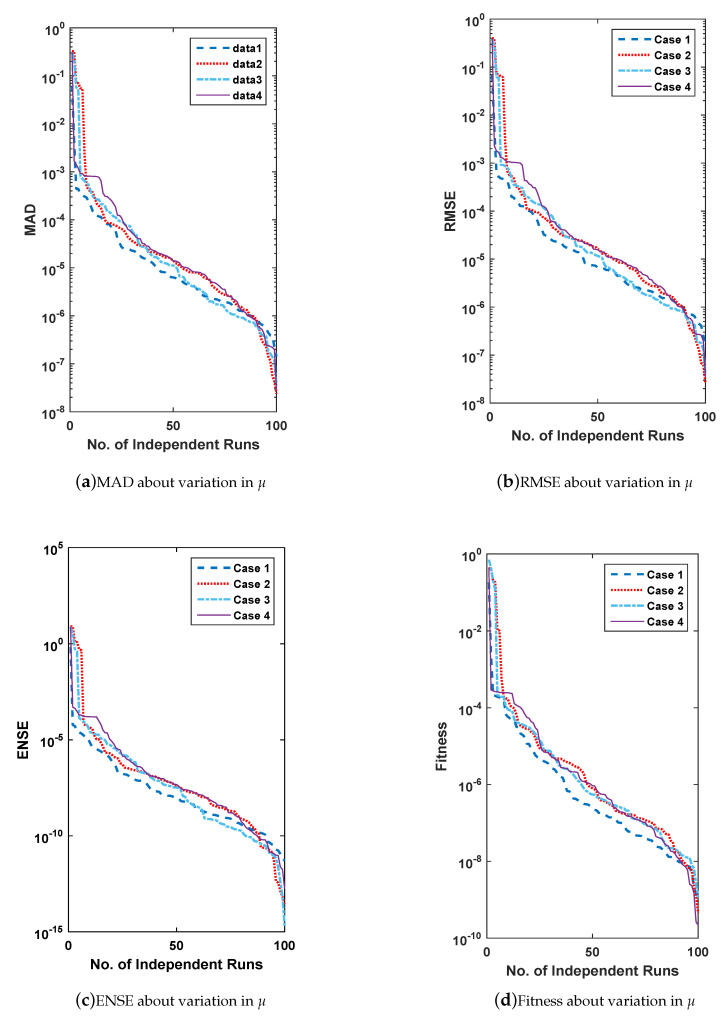
(**a**–**d**) show data of performance matrices for problem 2, based on the variation of μ. The data set is arranged in descending order and plot with a line having a log along the *y*-axis.

**Figure 12:**
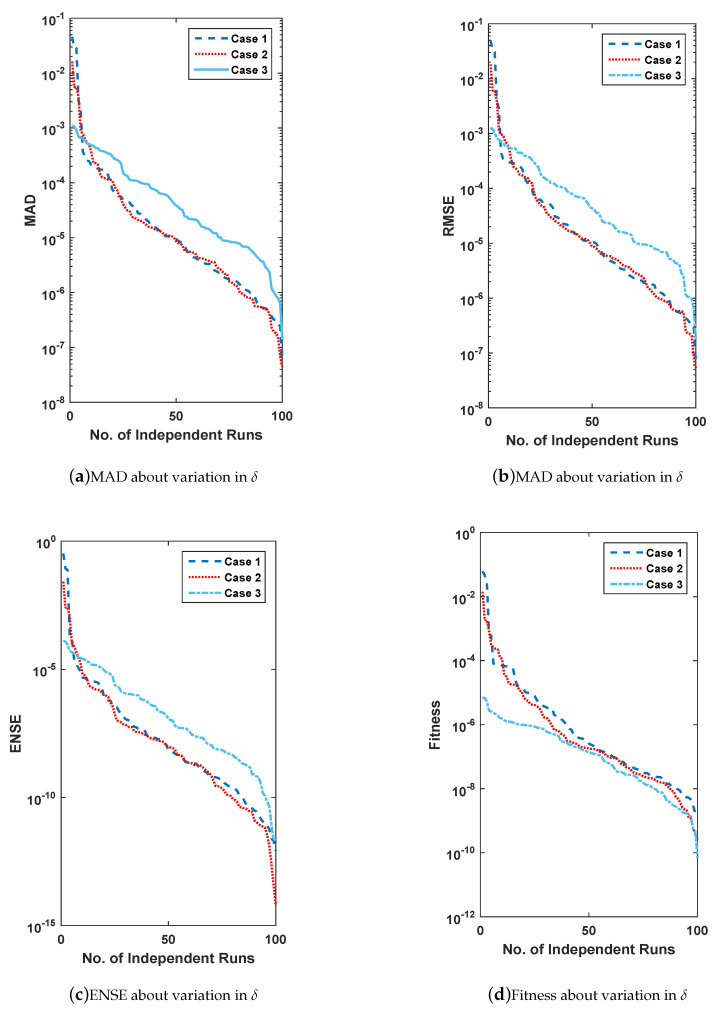
(**a**–**d**) show data of performance matrices for problem 3, based on the variation of δ. The data set is arranged in descending order and plot with a line having a log along the *y*-axis.

**Figure 13:**
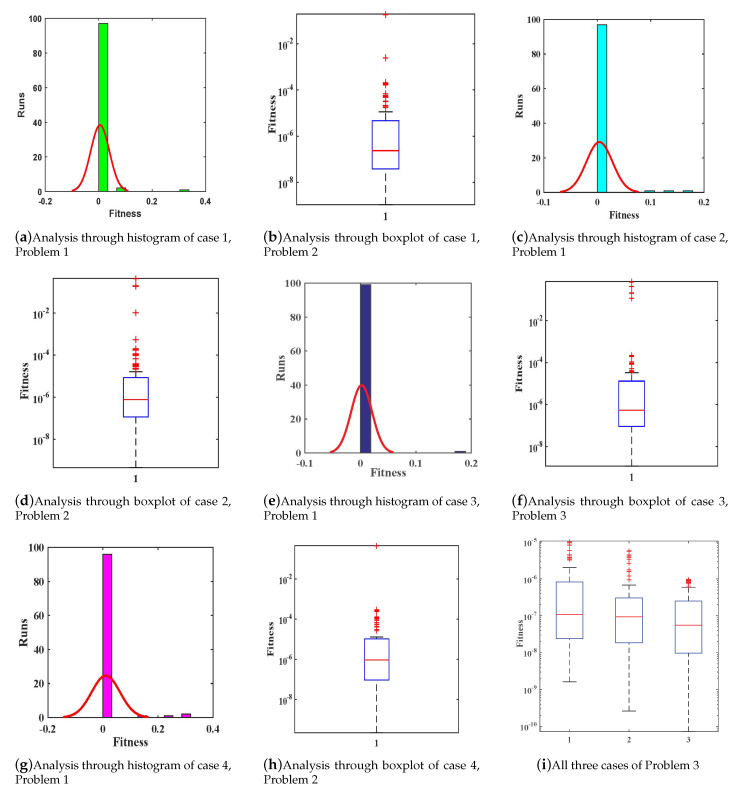
(**a**–**i**) show graphical analysis of fitness of all problems and their cases.

**Figure 14:**
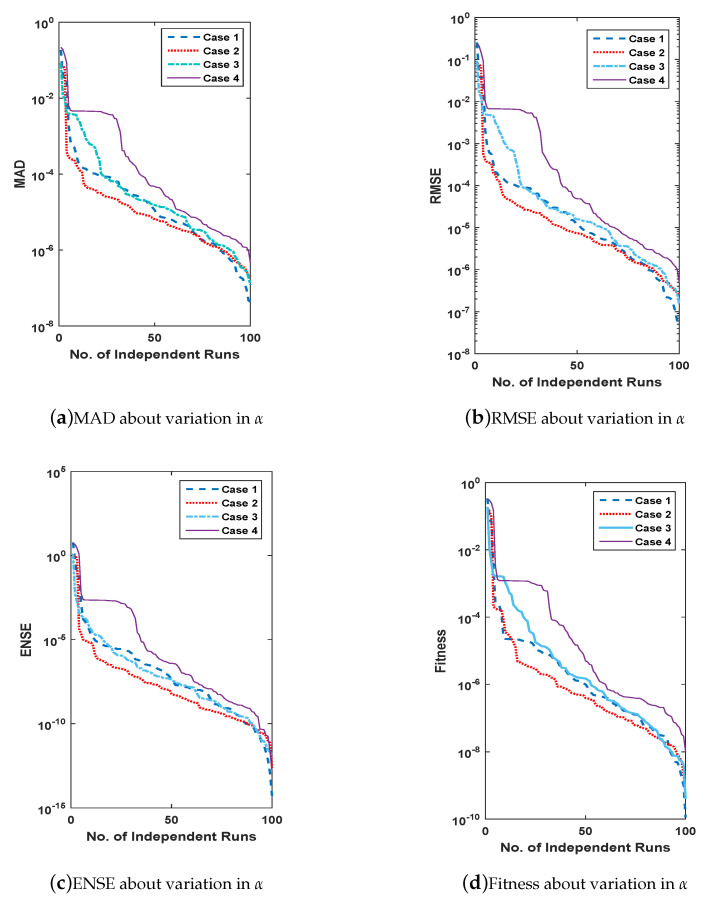
(**a**–**d**) show data of performance matrices for problem 1, based on the variation of α. The data set is arranged in descending order and plot with a line having a log along the *y*-axis.

For the evaluation of convergence and accuracy, 100 different runs are performed independently to find an appropriate set of unknowns or weights for all three problems of FSS. The sorted data of multiple runs of MAD, RMSE, ENSE and fitness are plotted in Figure 11, Figure 12, Figure 13 and Figure 14 for all problems of FSS, respectively. The graph semi-log-scale on the *y*-axis is used to clarify the small variation. The fitness of problems are also drawn with convergence plots given in Figure 13. The values of MAD, RMSE, ENSE, and the fitness of problem 1 are $10^{-6}$ to $10^{-8}$, $10^{-8}$ to $10^{-15}$, $10^{-6}$ to $10^{-8}$ and $10^{-6}$ to $10^{-10}$, values for problem 2 are $10^{-6}$ to $10^{-8}$, $10^{-8}$ to $10^{-15}$, $10^{-6}$ to $10^{-8}$ and $10^{-6}$ to $10^{-10}$, and values for problem 3 are $10^{-6}$ to $10^{-8}$, $10^{-8}$ to $10^{-15}$, $10^{-6}$ to $10^{-8}$ and $10^{-6}$ to $10^{-12}$ respectively. It seems that the values of global operators are comparatively better.

### 6.2. Analysis Based on Global Performance Indices

To evaluate the performance of proposed methodology through global indices, data are collected for 100 independent runs for the solution of all problems of FSS. The values of global operators with their means and standard deviation are given in Table 7. It is observed that values of these global operators are about $10^{-9}$ to $10^{-10}$, $10^{-9}$ to $10^{-10}$, $10^{-10}$ to $10^{-11}$, and $10^{-11}$ to $10^{-13}$ for GMAD, GRMSE, GENSE, and GFIT, respectively. Normally, the values of GFIT, GMAD, GENSE, and GRMSE express the consistency and reliability of the proposed scheme.

### 6.3. Complexity Analysis

For the performance of any technique, parameter setting is a key step. The ill parameters that can diverge affect the performance of a technique. In such a way, the performance of ANN-SCA-SQP algorithm is analyzed by variation of its parameter population and number of neurons. For the best performance number of neurons and population, 30 is taken. The results for all parameters are evaluated in terms of absolute error calculated using a reference solution of GA-ASM. The data for variation in population are reported in Table 8. The tuning of parameters is tested on all three problems for different cases. From the table, it seems that the absolute errors in solution of FSS for 20 population size are between $10^{-6}$ to $10^{-8}$; for a population size of 30, the errors lies between $10^{-8}$ to $10^{-10}$ and, for a population size of 40, the errors in solution are from $10^{-4}$ to $10^{-6}$, which verifies that the ANN-SCA-SQP algorithm has the best performance for a population size of 30.

The errors for variation in the number of neurons are reported in Table 9. The tuning of neurons is tested on all three problems for different cases. From the table, it seems that the absolute errors in the solution of FSS for 15 neurons are between $10^{-4}$ to $10^{-6}$, for a population size of 30, the errors lie between $10^{-8}$ to $10^{-10}$ and, for 45 neurons, the errors in solution are from $10^{-4}$ to $10^{-6}$, which verifies that the ANN-SCA-SQP algorithm has the best performance on 30 neurons.

**Figure 15:**
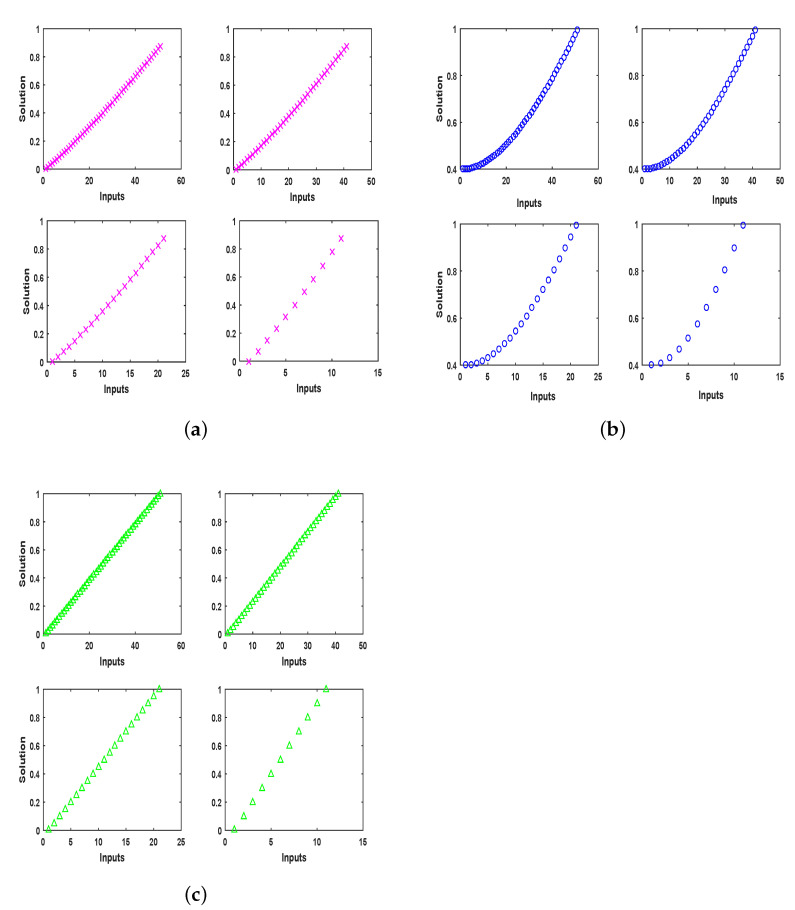
The graphs shows solution on input points 51, 41, 21, and 11, respectively. There is no effect of increasing or decreasing input points on solutions. (**a**) Problem 1: Solution for 51, 41, 21 and 11 input points; (**b**) Problem 2: Solution for 51, 41, 21 and 11 input points; (**c**) Problem 3: Solution for 51, 41, 21 and 11 input points.

The number of input points depend on the step size if input points vary between 0 and 1, if the step size is taken as 0.02, the interval will be split into 50 equal parts with 51 points and, if the step size is changed to 0.05, the points will change to 20, etc. The input points do not effect the performance of the proposed approach. All three problems are solved by execution of the proposed approach with 51, 41, 21, and 11 input points, and their results are drawn in Figure 15. It can clearly be observed that there is no effect on the solution except for a concentration of points.

## 7. Conclusions

We considered the celebrated nonlinear dynamic differential equation, known as the Falkner–Skan system, that arises in fluid dynamics for boundary-layer flow with the stream-wise pressure gradient transfer of mass over a dynamic wall. It has many applications like Falkner–Skan flow of chemically reactive cross nanofluid with heat generation/absorption and Falkner–Skan flow of Maxwell nanomaterials with heat and mass transfer over a static/moving wedge. To analyze dynamic characteristics of the boundary flow of the FS model, neurocomputing is utilized. An effective and robust neuro-stochastic computational solver is designed by the combination of unsupervised and supervised mechanisms by exploiting the worth of artificial neural networks with the help of Sine-Cosine Algorithm and Sequential Quadratic Programming. The numerical results found by the ANN-SCA-SQP algorithm are compared graphically as shown in Figure 5, Figure 7 and Figure 9, and numerically given in Table 1, Table 3 and Table 5, with the results of GA-ASM. The convergence and accuracy are verified by the consistent overlapping of solutions obtained by the proposed scheme with the reference solutions. For the evaluation of robustness of the designed methodology, different variants of the Falkner–Skan system based on the variation of wall stretching parameter δ, streamwise pressure gradient parameter α, and mass transfer at wall parameter μ are executed 100 times. The statistical evaluation based on 100 runs shows a small variation in values along with mean and standard variation. Different statistical performance measures were used i.e., MAD (Mean absolute deviation), RMSE (root mean square error), and ENSE (error in Nash–Sutcliffe efficiency) to analyze the performance of the proposed scheme. The global version of MAD, ENSE, and RMSE were also implemented along with their mean and standard deviation for reliability and effectiveness. Convergence plots were used for validation i.e., boxplot and histogram with normal distribution.

As there is a possibility that in the future other hybrid techniques may out perform the ANN-SCA-SQP algorithm, for further better accuracy and convergence, one may design a neural network-based artificial intelligence solver trained by a marine predator algorithm, particle swarm optimization, or other such evolutionary algorithms, etc., for the solution of variants of the Falkner–Skan system. The ANN-SCA-SQP algorithm can be implemented for other physical, complex, and biological problems.

## Figures and Tables

**Table 1:** Solution comparison of problem 1.

t	GA-ASM	ANN-SCA-SQP	GA-ASM	ANN-SCA-SQP	GA-ASM	ANN-SCA-SQP	GA-ASM	ANN-SCA-SQP
	**Case 1**		**Case 2**		**Case 3**		**Case 4**	
0	$3.29\times 10^{-9}$	$6.05\times 10^{-7}$	$6.53\times 10^{-9}$	$2.54\times 10^{-7}$	$4.08\times 10^{-9}$	$-5.47\times 10^{-7}$	$2.66\times 10^{-9}$	$1.65\times 10^{-6}$
0.1	0.005405388	0.005405935	0.00701195	0.007012223	0.008586938	0.008586741	0.011216733	0.011218571
0.2	0.021552704	0.021553204	0.027385678	0.027385963	0.033034377	0.033034394	0.042277563	0.042279561
0.3	0.048330482	0.048330938	0.060144874	0.060145167	0.071441575	0.071441658	0.089548039	0.089550157
0.4	0.085609334	0.085609743	0.104348531	0.104348845	0.122043285	0.12204328	0.149840904	0.149843023
0.5	0.133233738	0.1332341	0.159104966	0.159105304	0.183247294	0.183247067	0.220477782	0.220479818
0.6	0.191014645	0.191014961	0.223583479	0.223583826	0.253656044	0.253655481	0.299285991	0.299287959
0.7	0.25872314	0.258723423	0.297023781	0.297024106	0.332076339	0.332075319	0.384560326	0.384562273
0.8	0.336085496	0.336085755	0.378743274	0.378743557	0.417520623	0.417518995	0.475010319	0.475012229
0.9	0.422779921	0.422780154	0.468142379	0.468142625	0.509202753	0.509200337	0.56970587	0.569707661
1	0.518435328	0.51843553	0.564708054	0.564708269	0.606530566	0.606527172	0.66802863	0.668030264

**Table 2:** Statistical evaluation of problem 1 in terms of mean, minimum, and standard deviation.

t	α=0.1			α=1			α=2			α=4		
	**MIN**	**MEAN**	**STD**	**MIN**	**MEAN**	**STD**	**MIN**	**MEAN**	**STD**	**MIN**	**MEAN**	**STD**
0	$3.35\times 10^{-12}$	$1.450\times 10^{-8}$	$3.280\times 10^{-8}$	$5.04\times 10^{-12}$	$6.540\times 10^{-8}$	$1.150\times 10^{-7}$	$8.71\times 10^{-12}$	$1.280\times 10^{-8}$	$1.850\times 10^{-8}$	$3.39\times 10^{-10}$	$1.110\times 10^{-8}$	$9.70\times 10^{-9}$
0.1	$7.14\times 10^{-11}$	$4.460\times 10^{-8}$	$6.090\times 10^{-8}$	$3.74\times 10^{-12}$	$1.820\times 10^{-7}$	$2.280\times 10^{-7}$	$3.11\times 10^{-12}$	$5.230\times 10^{-8}$	$5.860\times 10^{-8}$	$1.36\times 10^{-9}$	$1.070\times 10^{-7}$	$7.390\times 10^{-8}$
0.2	$5.22\times 10^{-12}$	$2.590\times 10^{-8}$	$3.490\times 10^{-8}$	$1.34\times 10^{-107}$	$8.490\times 10^{-8}$	$1.190\times 10^{-7}$	$4.09\times 10^{-12}$	$2.350\times 10^{-8}$	$3.140\times 10^{-8}$	$1.67\times 10^{-10}$	$7.770\times 10^{-8}$	$1.190\times 10^{-7}$
0.3	$1.81\times 10^{-12}$	$2.270\times 10^{-8}$	$2.860\times 10^{-8}$	$3.20\times 10^{-12}$	$7.970\times 10^{-8}$	$1.330\times 10^{-7}$	$1.18\times 10^{-11}$	$2.550\times 10^{-8}$	$3.620\times 10^{-8}$	$2.86\times 10^{-9}$	$6.410\times 10^{-8}$	$5.980\times 10^{-8}$
0.4	$1.07\times 10^{-11}$	$1.510\times 10^{-8}$	$1.840\times 10^{-8}$	$4.30\times 10^{-11}$	$7.390\times 10^{-8}$	$9.850\times 10^{-8}$	$2.43\times 10^{-10}$	$2.670\times 10^{-8}$	$3.240\times 10^{-8}$	$2.08\times 10^{-9}$	$5.930\times 10^{-8}$	$6.140\times 10^{-8}$
0.5	$3.17\times 10^{-11}$	$1.830\times 10^{-8}$	$2.290\times 10^{-8}$	$1.07\times 10^{-11}$	$7.070\times 10^{-8}$	$9.870\times 10^{-8}$	$1.18\times 10^{-11}$	$2.180\times 10^{-8}$	$2.860\times 10^{-8}$	$4.40\times 10^{-9}$	$5.040\times 10^{-8}$	$6.180\times 10^{-8}$
0.6	$1.07\times 10^{-10}$	$2.950\times 10^{-8}$	$3.430\times 10^{-8}$	$7.04\times 10^{-11}$	$8.640\times 10^{-8}$	$1.340\times 10^{-7}$	$6.54\times 10^{-12}$	$2.480\times 10^{-8}$	$4.000\times 10^{-8}$	$1.67\times 10^{-10}$	$6.420\times 10^{-8}$	$6.070\times 10^{-8}$
0.7	$1.02\times 10^{-11}$	$1.600\times 10^{-8}$	$1.690\times 10^{-8}$	$2.23\times 10^{-12}$	$5.980\times 10^{-8}$	$9.070\times 10^{-8}$	$8.88\times 10^{-10}$	$2.280\times 10^{-8}$	$2.650\times 10^{-8}$	$1.410\times 10^{-8}$	$5.350\times 10^{-8}$	$4.470\times 10^{-8}$
0.8	$1.67\times 10^{-11}$	$2.590\times 10^{-8}$	$3.540\times 10^{-8}$	$3.23\times 10^{-12}$	$8.670\times 10^{-8}$	$1.080\times 10^{-7}$	$2.83\times 10^{-13}$	$1.910\times 10^{-8}$	$2.900\times 10^{-8}$	$5.18\times 10^{-10}$	$3.980\times 10^{-8}$	$4.730\times 10^{-8}$
0.9	$3.30\times 10^{-10}$	$6.770\times 10^{-8}$	$7.620\times 10^{-8}$	$6.72\times 10^{-11}$	$1.790\times 10^{-7}$	$2.460\times 10^{-7}$	$1.75\times 10^{-10}$	$5.980\times 10^{-8}$	$7.730\times 10^{-8}$	$2.730\times 10^{-8}$	$1.150\times 10^{-7}$	$9.260\times 10^{-8}$
1	$1.40\times 10^{-11}$	$3.210\times 10^{-8}$	$5.380\times 10^{-8}$	$2.08\times 10^{-11}$	$1.120\times 10^{-7}$	$1.560\times 10^{-7}$	$1.04\times 10^{-12}$	$2.890\times 10^{-8}$	$4.380\times 10^{-8}$	$2.23\times 10^{-10}$	$3.36\times 10^{-8}$	$4.97\times 10^{-8}$

**Table 3:** Solution comparison of problem 2.

t	GA-ASM	ANN-SCA-SQP	GA-ASM	ANN-SCA-SQP	GA-ASM	ANN-SCA-SQP	GA-ASM	ANN-SCA-SQP
	**Case 1**		**Case 2**		**Case 3**		**Case 4**	
0.922, 0.945, 0.871 0	0.100000003	0.10000286	0.400000001	0.40000002	0.700000002	0.70000006	1.000000002	1.00000916
0.1	0.107258688	0.10726099	0.408030229	0.40803026	0.708845878	0.7088459	1.009701424	1.00971087
0.922, 0.945, 0.871 0.2	0.12827934	0.12828114	0.431049908	0.43104994	0.733940924	0.73394093	1.036933177	1.03694284
0.3	0.161955186	0.16195656	0.467521105	0.46752114	0.773256408	0.77325642	1.079117187	1.07912697
0.922, 0.945, 0.871 0.4	0.207227704	0.2072287	0.516009591	0.51600963	0.824950845	0.82495083	1.133977968	1.13398776
0.5	0.263100533	0.26310118	0.575196183	0.57519622	0.88737351	0.88737345	1.199530471	1.19954018
0.922, 0.945, 0.871 0.6	0.32865057	0.32865088	0.643883848	0.64388387	0.959062703	0.95906263	1.274062714	1.27407226
0.7	0.403036431	0.40303644	0.721001173	0.72100119	1.038739877	1.03873981	1.356114951	1.35612425
0.922, 0.945, 0.871 0.8	0.485504502	0.48550426	0.805602746	0.80560276	1.125300632	1.12530058	1.444456808	1.44446581
0.9	0.575392805	0.57539238	0.896866986	0.89686699	1.217803468	1.21780345	1.53806357	1.53807222
0.922, 0.945, 0.871 1	0.672132912	0.67213233	0.994091896	0.99409188	1.315457047	1.31545705	1.636092513	1.63610078

**Table 4:** Statistical evaluation of problem 2 in terms of minimum, mean, and standard deviation.

t	Case 1			Case 2			Case 3			Case 4		
	**MIN**	**MEAN**	**STD**	**MIN**	**MEAN**	**STD**	**MIN**	**MEAN**	**STD**	**MIN**	**MEAN**	**STD**
0	$1.24\times 10^{-11}$	$2.99\times 10^{-8}$	$6.60\times 10^{-8}$	$1.99\times 10^{-12}$	$1.57\times 10^{-7}$	$2.59\times 10^{-7}$	$6.55\times 10^{-13}$	$1.17\times 10^{-7}$	$1.88\times 10^{-7}$	$3.01\times 10^{-11}$	$7.98\times 10^{-8}$	$1.67\times 10^{-7}$
0.1	$2.36\times 10^{-10}$	$1.02\times 10^{-7}$	$1.37\times 10^{-7}$	$1.74\times 10^{-10}$	$3.43\times 10^{-7}$	$3.83\times 10^{-7}$	$8.34\times 10^{-11}$	$3.37\times 10^{-7}$	$4.27\times 10^{-7}$	$2.67\times 10^{-12}$	$3.18\times 10^{-7}$	$4.55\times 10^{-7}$
0.2	$3.40\times 10^{-11}$	$7.20\times 10^{-8}$	$1.43\times 10^{-7}$	$7.00\times 10^{-13}$	$1.53\times 10^{-7}$	$2.61\times 10^{-7}$	$1.14\times 10^{-11}$	$1.04\times 10^{-7}$	$1.79\times 10^{-7}$	$2.31\times 10^{-12}$	$1.44\times 10^{-7}$	$2.49\times 10^{-7}$
0.3	$1.29\times 10^{-11}$	$6.25\times 10^{-8}$	$9.65\times 10^{-8}$	$3.62\times 10^{-11}$	$9.67\times 10^{-8}$	$1.13\times 10^{-7}$	$2.47\times 10^{-11}$	$1.21\times 10^{-7}$	$1.82\times 10^{-7}$	$5.35\times 10^{-12}$	$1.58\times 10^{-7}$	$2.37\times 10^{-7}$
0.4	$2.07\times 10^{-11}$	$4.45\times 10^{-8}$	$6.94\times 10^{-8}$	$2.53\times 10^{-10}$	$1.52\times 10^{-7}$	$2.02\times 10^{-7}$	$2.81\times 10^{-11}$	$1.20\times 10^{-7}$	$1.44\times 10^{-7}$	$6.32\times 10^{-13}$	$1.23\times 10^{-7}$	$2.50\times 10^{-7}$
0.5	$4.92\times 10^{-11}$	$5.01\times 10^{-8}$	$7.39\times 10^{-8}$	$4.85\times 10^{-11}$	$1.26\times 10^{-7}$	$2.35\times 10^{-7}$	$5.76\times 10^{-11}$	$9.82\times 10^{-8}$	$1.54\times 10^{-7}$	$2.07\times 10^{-12}$	$1.47\times 10^{-7}$	$2.15\times 10^{-7}$
0.6	$1.17\times 10^{-10}$	$6.17\times 10^{-8}$	$8.34\times 10^{-8}$	$7.45\times 10^{-12}$	$1.00\times 10^{-7}$	$1.35\times 10^{-7}$	$5.42\times 10^{-12}$	$1.09\times 10^{-7}$	$1.46\times 10^{-7}$	$6.89\times 10^{-13}$	$1.13\times 10^{-7}$	$1.86\times 10^{-7}$
0.7	$1.74\times 10^{-11}$	$3.69\times 10^{-8}$	$5.93\times 10^{-8}$	$3.03\times 10^{-11}$	$9.03\times 10^{-8}$	$1.24\times 10^{-7}$	$7.69\times 10^{-11}$	$8.60\times 10^{-8}$	$1.10\times 10^{-7}$	$1.32\times 10^{-11}$	$1.14\times 10^{-7}$	$2.64\times 10^{-7}$
0.8	$5.05\times 10^{-11}$	$5.04\times 10^{-8}$	$6.18\times 10^{-8}$	$1.74\times 10^{-11}$	$1.19\times 10^{-7}$	$2.21\times 10^{-7}$	$2.00\times 10^{-12}$	$1.25\times 10^{-7}$	$1.96\times 10^{-7}$	$2.69\times 10^{-11}$	$1.74\times 10^{-7}$	$3.58\times 10^{-7}$
0.9	$9.22\times 10^{-10}$	$1.08\times 10^{-7}$	$1.27\times 10^{-7}$	$3.46\times 10^{-10}$	$2.02\times 10^{-7}$	$2.45\times 10^{-7}$	$8.04\times 10^{-10}$	$2.36\times 10^{-7}$	$2.71\times 10^{-7}$	$1.65\times 10^{-12}$	$2.51\times 10^{-7}$	$4.53\times 10^{-7}$
1	$3.15\times 10^{-14}$	$6.44\times 10^{-8}$	$8.26\times 10^{-8}$	$1.19\times 10^{-12}$	$1.33\times 10^{-7}$	$2.12\times 10^{-7}$	$1.13\times 10^{-11}$	$1.77\times 10^{-7}$	$2.43\times 10^{-7}$	$1.65\times 10^{-13}$	$2.25\times 10^{-7}$	$4.73\times 10^{-7}$

**Table 5:** Solution comparison of problem 3.

t	GA-ASM	ANN-SCA-SQP	GA-ASM	ANN-SCA-SQP	GA-ASM	ANN-SCA-SQP
0.886, 0.937, 0.855	**Case 1**		**Case 2**		**Case 3**	
0	$1.82\times 10^{-9}$	$8.36\times 10^{-7}$	$-3.26\times 10^{-10}$	$-8.28\times 10^{-7}$	0.00000000201791	−0.00000000634108
0.886, 0.937, 0.855 0.1	0.04453269581415	0.04453360804663	0.07238126197681	0.07238058398271	0.10000000050947	0.09999997846340
0.2	0.09759136204994	0.09759233310773	0.14920280312497	0.14920225200297	0.20000000004858	0.19999995379779
0.886, 0.937, 0.855 0.3	0.15840171232390	0.15840276011892	0.23000887771757	0.23000841060721	0.30000000060035	0.29999992810436
0.4	0.22624072485599	0.22624184509193	0.31438156183320	0.31438112523451	0.39999999987835	0.39999990191691
0.886, 0.937, 0.855 0.5	0.30044143682626	0.30044259729796	0.40194144929501	0.40194100409706	0.49999999785942	0.49999987338517
0.6	0.38039632386608	0.38039747408055	0.49234775039453	0.49234728211637	0.59999999685985	0.59999984254489
0.886, 0.937, 0.855 0.7	0.46555936972989	0.46556045397667	0.58529783585290	0.58529734522883	0.69999999814024	0.69999981035180
0.8	0.55544695545873	0.55544793048215	0.68052628070707	0.68052576767795	0.79999999993310	0.79999977481840
0.886, 0.937, 0.855 0.9	0.64963770195136	0.64963855179176	0.77780347991899	0.77780293432006	0.89999999991166	0.89999972856515
1	0.74777138206612	0.74777211254454	0.87693391214263	0.87693331575639	0.99999999945938	0.99999966257045

**Table 6:** Statistical evaluation of problem 3 in terms of minimum, mean, and standard deviation.

t	Case 1			Case 2			Case 3		
	**MIN**	**MEAN**	**STD**	**MIN**	**MEAN**	**STD**	**MIN**	**MEAN**	**STD**
0	$1.82\times 10^{-11}$	$1.09\times 10^{-8}$	$1.20\times 10^{-8}$	$7.18\times 10^{-11}$	$5.41\times 10^{-8}$	$8.06\times 10^{-8}$	$7.18\times 10^{-11}$	$7.34\times 10^{-9}$	$9.28\times 10^{-9}$
0.1	$3.65\times 10^{-10}$	$3.15\times 10^{-8}$	$3.21\times 10^{-8}$	$1.93\times 10^{-11}$	$8.45\times 10^{-8}$	$1.01\times 10^{-7}$	$1.93\times 10^{-11}$	$2.26\times 10^{-8}$	$2.17\times 10^{-8}$
0.2	$1.51\times 10^{-11}$	$8.78\times 10^{-9}$	$9.38\times 10^{-9}$	$2.37\times 10^{-12}$	$5.82\times 10^{-8}$	$1.11\times 10^{-7}$	$2.37\times 10^{-12}$	$7.99\times 10^{-9}$	$1.29\times 10^{-8}$
0.3	$7.06\times 10^{-12}$	$1.10\times 10^{-8}$	$1.54\times 10^{-8}$	$1.44\times 10^{-12}$	$2.69\times 10^{-8}$	$5.82\times 10^{-8}$	$1.44\times 10^{-12}$	$8.01\times 10^{-9}$	$1.00\times 10^{-8}$
0.4	$1.55\times 10^{-11}$	$1.33\times 10^{-8}$	$1.47\times 10^{-8}$	$2.46\times 10^{-10}$	$3.81\times 10^{-8}$	$5.19\times 10^{-8}$	$2.46\times 10^{-10}$	$8.97\times 10^{-9}$	$1.01\times 10^{-8}$
0.5	$1.26\times 10^{-11}$	$1.02\times 10^{-8}$	$9.96\times 10^{-9}$	$6.19\times 10^{-14}$	$4.39\times 10^{-8}$	$6.97\times 10^{-8}$	$6.19\times 10^{-14}$	$7.01\times 10^{-9}$	$9.94\times 10^{-9}$
0.6	$5.43\times 10^{-12}$	$8.22\times 10^{-9}$	$1.14\times 10^{-8}$	$1.55\times 10^{-12}$	$2.81\times 10^{-8}$	$4.23\times 10^{-8}$	$1.55\times 10^{-12}$	$7.39\times 10^{-9}$	$9.72\times 10^{-9}$
0.7	$6.89\times 10^{-13}$	$1.07\times 10^{-8}$	$1.44\times 10^{-8}$	$1.77\times 10^{-11}$	$2.76\times 10^{-8}$	$4.53\times 10^{-8}$	$1.77\times 10^{-11}$	$6.01\times 10^{-9}$	$7.67\times 10^{-9}$
0.8	$1.69\times 10^{-10}$	$1.08\times 10^{-8}$	$1.22\times 10^{-8}$	$2.00\times 10^{-10}$	$5.45\times 10^{-8}$	$7.62\times 10^{-8}$	$2.00\times 10^{-10}$	$1.00\times 10^{-8}$	$1.05\times 10^{-8}$
0.9	$3.96\times 10^{-12}$	$2.42\times 10^{-8}$	$3.33\times 10^{-8}$	$1.24\times 10^{-13}$	$5.06\times 10^{-8}$	$7.90\times 10^{-8}$	$1.24\times 10^{-13}$	$1.30\times 10^{-8}$	$1.65\times 10^{-8}$
1	$7.47\times 10^{-11}$	$1.47\times 10^{-8}$	$1.64\times 10^{-8}$	$1.36\times 10^{-10}$	$6.32\times 10^{-8}$	$8.62\times 10^{-8}$	$1.36\times 10^{-10}$	$1.25\times 10^{-8}$	$1.09\times 10^{-8}$

**Table 7:** Statistical data of global operators in terms of minimum, mean, and standard deviation.

Problems	Cases	GMAD				GRMSE				GENSE				GFIT		
		MIN	MEAN	STD		MIN	MEAN	STD		MIN	MEAN	STD		MIN	MEAN	STD
1	1	$4.36\times 10^{-10}$	$1.86\times 10^{-8}$	$2.45\times 10^{-8}$		$4.77\times 10^{-10}$	$1.98\times 10^{-8}$	$2.62\times 10^{-8}$		$5.33\times 10^{-17}$	$3.46\times 10^{-11}$	$7.14\times 10^{-11}$		$1.12\times 10^{-12}$	$2.84\times 10^{-10}$	$2.34\times 10^{-10}$
	2	$1.98\times 10^{-9}$	$5.68\times 10^{-8}$	$9.59\times 10^{-8}$		$2.43\times 10^{-9}$	$5.83\times 10^{-8}$	$9.73\times 10^{-8}$		$2.49\times 10^{-15}$	$3.82\times 10^{-10}$	$1.05\times 10^{-9}$		$4.31\times 10^{-12}$	$3.56\times 10^{-10}$	$2.63\times 10^{-10}$
	3	$1.20\times 10^{-9}$	$4.48\times 10^{-8}$	$5.96\times 10^{-8}$		$1.47\times 10^{-9}$	$4.73\times 10^{-8}$	$6.06\times 10^{-8}$		$1.60\times 10^{-14}$	$1.45\times 10^{-10}$	$3.15\times 10^{-10}$		$3.98\times 10^{-12}$	$2.90\times 10^{-10}$	$2.69\times 10^{-10}$
	4	$4.62\times 10^{-9}$	$4.54\times 10^{-8}$	$3.50\times 10^{-8}$		$5.01\times 10^{-9}$	$4.63\times 10^{-8}$	$3.49\times 10^{-8}$		$4.74\times 10^{-13}$	$6.98\times 10^{-11}$	$8.78\times 10^{-11}$		9.98 × 10^−11^	6.15 × 10^−10^	3.57 × 10^−10^
2	1	$1.41\times 10^{-9}$	$6.52\times 10^{-8}$	$1.34\times 10^{-7}$		$1.45\times 10^{-9}$	$6.85\times 10^{-8}$	$1.38\times 10^{-7}$		$6.13\times 10^{-14}$	$6.35\times 10^{-10}$	$2.35\times 10^{-9}$		$1.09\times 10^{-11}$	$2.01\times 10^{-10}$	$1.46\times 10^{-10}$
	2	$2.40\times 10^{-10}$	$3.55\times 10^{-8}$	$6.97\times 10^{-8}$		$2.61\times 10^{-10}$	$3.66\times 10^{-8}$	$7.12\times 10^{-8}$		$2.50\times 10^{-16}$	$1.66\times 10^{-10}$	$6.13\times 10^{-10}$		$4.49\times 10^{-12}$	$2.69\times 10^{-10}$	$2.50\times 10^{-10}$
	3	$3.55\times 10^{-10}$	$5.16\times 10^{-8}$	$1.15\times 10^{-7}$		$4.36\times 10^{-10}$	$5.42\times 10^{-8}$	$1.21\times 10^{-7}$		$7.09\times 10^{-16}$	$4.05\times 10^{-10}$	$1.47\times 10^{-9}$		$1.14\times 10^{-11}$	$2.78\times 10^{-10}$	$2.33\times 10^{-10}$
	4	$2.82\times 10^{-10}$	$4.28\times 10^{-8}$	$5.52\times 10^{-8}$		$3.49\times 10^{-10}$	$4.40\times 10^{-8}$	$5.49\times 10^{-8}$		$1.78\times 10^{-15}$	$1.16\times 10^{-10}$	$2.53\times 10^{-10}$		$2.18\times 10^{-12}$	$1.58\times 10^{-10}$	$1.36\times 10^{-10}$
3	1	$6.61\times 10^{-10}$	$4.24\times 10^{-8}$	$7.08\times 10^{-8}$		$7.37\times 10^{-10}$	$4.55\times 10^{-8}$	$7.81\times 10^{-8}$		$8.34\times 10^{-15}$	$9.58\times 10^{-11}$	$3.02\times 10^{-10}$		$1.63\times 10^{-11}$	$1.43\times 10^{-10}$	$9.40\times 10^{-11}$
	2	$4.47\times 10^{-10}$	$3.61\times 10^{-8}$	$5.70\times 10^{-8}$		$5.33\times 10^{-10}$	$3.69\times 10^{-8}$	$5.71\times 10^{-8}$		$3.94\times 10^{-16}$	$6.24\times 10^{-11}$	$1.78\times 10^{-10}$		$2.61\times 10^{-12}$	$1.01\times 10^{-10}$	$8.05\times 10^{-11}$
	3	$1.41\times 10^{-9}$	$7.11\times 10^{-8}$	$7.12\times 10^{-8}$		$1.73\times 10^{-9}$	$7.73\times 10^{-8}$	$7.78\times 10^{-8}$		$2.18\times 10^{-14}$	$9.98\times 10^{-11}$	$2.29\times 10^{-10}$		$7.29\times 10^{-13}$	$5.18\times 10^{-11}$	$4.33\times 10^{-11}$

**Table 8:** Analysis of ANN-SCA-SQP by variation of population size.

Absolute Errors for Input Values η												
**Problem/Case**	**Population**	η=0	η=0.1	η=0.2	η=0.3	η=0.4	η=0.5	η=0.6	η=0.7	η=0.8	η=0.9	η=1.0
1	20	$7.89\times 10^{-7}$	$3.78\times 10^{-7}$	$1.50\times 10^{-7}$	$3.22\times 10^{-7}$	$4.62\times 10^{-8}$	$7.30\times 10^{-7}$	$1.31\times 10^{-6}$	$1.46\times 10^{-6}$	$1.20\times 10^{-6}$	$8.37\times 10^{-7}$	$8.04\times 10^{-7}$
	30	$1.47\times 10^{-7}$	$9.31\times 10^{-8}$	$2.00\times 10^{-8}$	$8.83\times 10^{-9}$	$4.14\times 10^{-8}$	$4.45\times 10^{-8}$	$3.50\times 10^{-10}$	$4.19\times 10^{-8}$	$3.58\times 10^{-8}$	$2.98\times 10^{-8}$	$5.08\times 10^{-8}$
	40	$3.43\times 10^{-5}$	$4.18\times 10^{-5}$	$4.39\times 10^{-5}$	$4.21\times 10^{-5}$	$3.72\times 10^{-5}$	$2.89\times 10^{-5}$	$1.56\times 10^{-5}$	$4.61\times 10^{-6}$	$3.34\times 10^{-5}$	$7.16\times 10^{-5}$	0.000119
2	20	$6.16\times 10^{-5}$	$1.09\times 10^{-5}$	$5.36\times 10^{-6}$	$2.44\times 10^{-5}$	0.00011	0.000254	0.000447	0.000678	0.000931	0.001198	0.001482
	30	$2.35\times 10^{-8}$	$3.07\times 10^{-8}$	$2.92\times 10^{-8}$	$3.01\times 10^{-8}$	$3.89\times 10^{-8}$	$3.75\times 10^{-8}$	$2.50\times 10^{-8}$	$1.65\times 10^{-8}$	$1.51\times 10^{-8}$	$5.67\times 10^{-9}$	$1.19\times 10^{-8}$
	40	$8.93\times 10^{-7}$	$4.25\times 10^{-7}$	$1.51\times 10^{-7}$	$4.82\times 10^{-7}$	$5.15\times 10^{-7}$	$4.75\times 10^{-7}$	$6.27\times 10^{-7}$	$1.07\times 10^{-6}$	$1.69\times 10^{-6}$	$2.30\times 10^{-6}$	$2.74\times 10^{-6}$
3	20	$7.89\times 10^{-7}$	$3.78\times 10^{-7}$	$1.50\times 10^{-7}$	$3.22\times 10^{-7}$	$4.62\times 10^{-8}$	$7.30\times 10^{-7}$	$1.31\times 10^{-6}$	$1.46\times 10^{-6}$	$1.20\times 10^{-6}$	$8.37\times 10^{-7}$	$8.04\times 10^{-7}$
	30	$2.83\times 10^{-8}$	$3.89\times 10^{-8}$	$4.10\times 10^{-8}$	$1.47\times 10^{-8}$	$1.14\times 10^{-8}$	3.96 × 10^−9^	$3.74\times 10^{-8}$	$8.12\times 10^{-8}$	$9.40\times 10^{-8}$	$7.62\times 10^{-8}$	$6.50\times 10^{-8}$
	40	$3.43\times 10^{-5}$	$4.18\times 10^{-5}$	$4.39\times 10^{-5}$	$4.21\times 10^{-5}$	$3.72\times 10^{-5}$	$2.89\times 10^{-5}$	$1.56\times 10^{-5}$	$4.61\times 10^{-6}$	$3.34\times 10^{-5}$	$7.16\times 10^{-5}$	0.000119

**Table 9:** Analysis of ANN-SCA-SQP by variation in number of neurons.

Absolute Errors for Input Values η												
**Problem/Case**	**No. of Neurons**	η=0	η=1	η=0.2	η=0.3	η=0.4	η=0.5	η=0.6	η=0.7	η=0.8	η=0.9	η=1.0
**1**	**15**	$8.80\times 10^{-6}$	$9.21\times 10^{-6}$	$1.01\times 10^{-5}$	$9.97\times 10^{-6}$	$8.67\times 10^{-6}$	$7.13\times 10^{-6}$	$6.80\times 10^{-6}$	$8.63\times 10^{-6}$	$1.23\times 10^{-5}$	$1.64\times 10^{-5}$	$1.92\times 10^{-5}$
	**30**	1.47 × 10^−7^	$9.31\times 10^{-8}$	$2.00\times 10^{-8}$	$8.83\times 10^{-9}$	$4.14\times 10^{-8}$	$4.45\times 10^{-8}$	$3.50\times 10^{-10}$	$4.19\times 10^{-8}$	$3.58\times 10^{-8}$	$2.98\times 10^{-8}$	$5.08\times 10^{-8}$
	**45**	0.000937	0.044811	0.079813	0.10418	0.118043	0.121557	0.11491	0.098331	0.072095	0.036523	0.008015
**2**	**15**	0.000133	0.000144	0.000149	0.000152	0.000155	0.000158	0.000158	0.000152	0.000138	0.000117	$9.10\times 10^{-5}$
	**30**	$2.35\times 10^{-8}$	$3.07\times 10^{-8}$	$2.92\times 10^{-8}$	$3.01\times 10^{-8}$	$3.89\times 10^{-8}$	$3.75\times 10^{-8}$	$2.50\times 10^{-8}$	$1.65\times 10^{-8}$	$1.51\times 10^{-8}$	$5.67\times 10^{-9}$	$1.19\times 10^{-8}$
	**45**	$2.01\times 10^{-5}$	$1.84\times 10^{-5}$	$1.76\times 10^{-5}$	$1.84\times 10^{-5}$	$1.90\times 10^{-5}$	$1.82\times 10^{-5}$	$1.61\times 10^{-5}$	$1.40\times 10^{-5}$	$1.33\times 10^{-5}$	$1.38\times 10^{-5}$	$1.41\times 10^{-5}$
**3**	**15**	$1.28\times 10^{-5}$	$2.82\times 10^{-5}$	$3.54\times 10^{-5}$	$3.05\times 10^{-5}$	$1.03\times 10^{-5}$	$2.59\times 10^{-5}$	$7.61\times 10^{-5}$	0.000137	0.000203	0.000274	0.00035
	**30**	$2.83\times 10^{-8}$	$3.89\times 10^{-8}$	$4.10\times 10^{-8}$	$1.47\times 10^{-8}$	$1.14\times 10^{-8}$	$3.96\times 10^{-9}$	$3.74\times 10^{-8}$	$8.12\times 10^{-8}$	$9.40\times 10^{-8}$	$7.62\times 10^{-8}$	$6.50\times 10^{-8}$
	**45**	$1.26\times 10^{-5}$	$1.26\times 10^{-5}$	$1.25\times 10^{-5}$	$1.19\times 10^{-5}$	$1.09\times 10^{-5}$	$9.77\times 10^{-6}$	$8.88\times 10^{-6}$	$8.45\times 10^{-6}$	$8.30\times 10^{-6}$	$7.99\times 10^{-6}$	$7.13\times 10^{-6}$

## Data Availability

The data that support the findings of this study are available from the corresponding author upon reasonable request.

## Abbreviations

The following abbreviations are used in this manuscript:

ϕ	Activation function
$\hat{g}\left(\eta \right)$	Approximate ANN based Solution
GA	Genetic Algorithm
ASM	Active Set Method
FSS	Falkner–Skan System
SCA	Sine-Cosine Algorithm
STD	Standard Deviation
SQP	Sequential Quadratic Programming
ANN	Artificial Neural Network
MAD	Mean-Absolute Derivation
RMSE	Root-Mean Square Error
ENSE	Error in Nash-Sutcliffe Efficiency

## Appendix A

Approximated solution of problem 1 for all cases:

(A1) $$\begin{array}{c} g_{c1}\left(\eta \right)=\frac{7.87836342162768}{1+e^{-(0.886012386992085\eta -1.72218406243382)}}+\frac{-3.94998347150690}{1+e^{-(-9.57893780202074\eta -22.8166219837040)}}\\ +\frac{0.00682256641931040}{1+e^{-(5.09999272865296\eta -8.27285659877981)}}+\frac{-12.1752100238480}{1+e^{-(-13.2437845409868\eta -25.5427775843190)}}\\ +\frac{17.3889383163753}{1+e^{-(-1.09118478653714\eta -3.77090407319657)}}+\frac{-0.0350811277376602}{1+e^{-(0.0732626841290201\eta +13.4437840186043)}}\\ +\frac{4.73957108812539}{1+e^{-(6.99993786026066\eta -19.3910131716001)}}+\frac{-0.200582444909643}{1+e^{-(6.99993786026066\eta +24.9266845869495)}}\\ +\frac{-2.21758180223300}{1+e^{-(0.909233858204770\eta +0.442323650910280)}}+\frac{-19.4404271466426}{1+e^{-9.97392865949384\eta -19.2287655090247)}}, \end{array}$$

(A2) $$\begin{array}{c} g_{c2}\left(\eta \right)=\frac{5.14035320423249}{1+e^{-(1.25191046826097\eta -4.73830687416967}}+\frac{-6.73678252541636}{1+e^{-(-29.9925754469261\eta -19.1042795441711)}}\\ +\frac{-5.97597929880291}{1+e^{-(1.25739897207087\eta +2.08216581269597)}}+\frac{3.08370403155240}{1+e^{-0.449936184456119\eta -7.63512121228088)}}\\ +\frac{19.2240727364342}{1+e^{-(-0.853668038542871\eta -3.16457109971809)}}+\frac{25.3596943911278}{1+e^{0.202277070705441\eta -1.41618253668304)}}\\ +\frac{-1.43402682686793}{1+e^{-(9.31120486775986\eta +13.3096048760497)}}+\frac{10.4884971736156}{1+e^{-0.181130123759785\eta -17.6546052866385)}}\\ +\frac{2.11473522165581}{1+e^{-(0.986803092873646\eta -0.164471026440938)}}+\frac{6.20343683446747}{1+e^{-1.66637382492571\eta -10.4633659927988)}}, \end{array}$$

(A3) $$\begin{array}{c} g_{c3}\left(\eta \right)=\frac{9.89100631955405}{1+e^{-(6.60991428725725\eta -29.8573995984460}}+\frac{12.3422899288533}{1+e^{-(0.0286765754238881\eta -10.9005621648372)}}\\ +\frac{8.85432754858503}{1+e^{-(1.52523445812372\eta -5.79065021027889)}}+\frac{-3.35038587409801}{1+e^{-(-0.0544681171849533\eta -8.92924892097382)}}\\ +\frac{-2.05439995377823}{1+e^{-(-3.43807114396258\eta +29.525004742456)}}+\frac{19.1852783176006}{1+e^{-(-1.59819194279389\eta -2.28580265311327)}}\\ +\frac{3.90987926073757}{1+e^{-(1.44068174566448\eta -24.1265843865795)}}+\frac{12.8568792663011}{1+e^{-(0.252891071716880\eta -0.115633528840610)}}\\ +\frac{-4.17169381490917}{1+e^{-(13.0988996250089\eta 23.8723213491371)}}+\frac{-18.6040871901027}{1+e^{-(-1.15587734783564\eta -2.34394343145133)}}, \end{array}$$

(A4) $$\begin{array}{c} g_{c4}\left(\eta \right)=\frac{-2.25715619880877}{1+e^{-(1.17752273972245\eta +0.0490323818612627}}+\frac{-4.19597307635984}{1+e^{-(8.60059818955635\eta -30.0000005031497)}}\\ +\frac{5.81645052069167}{1+e^{-(-2.59893906954114\eta -2.26873022584258)}}+\frac{11.7088601742965}{1+e^{-(1.51146214483898\eta -12.2708440738918)}}\\ +\frac{12.8467185768014}{1+e^{-(0.663995785861310\eta +0.651649438268842)}}+\frac{20.9552891374767}{1+e^{-(5.80033109081801\eta +13.2754609635146)}}\\ +\frac{-1.32893471172670}{1+e^{-(20.9229180711128\eta +16.7231244899465)}}+\frac{-17.4312667850442}{1+e^{-(7.32178292444245\eta -31.4438293886643)}}\\ +\frac{-27.0939021314063}{1+e^{-(-1.77687446776186\eta +7.49257295303940)}}+\frac{3.81418567176834}{1+e^{-(-3.35646298701331\eta +14.9105595398540)}}. \end{array}$$

Approximated solution of problem 2 for all cases:

(A5) $$\begin{array}{c} g_{c1}\left(\eta \right)=\frac{0.393186143204226}{1+e^{-(-15.4530561261540\eta -28.1365682325099}}+\frac{14.1327028965341}{1+e^{-(-1.16184774595146\eta -2.34607039789879)}}\\ +\frac{-12.7575876390472}{1+e^{-(-0.687465779767490\eta +3.28197571046131)}}+\frac{4.72954133684452}{1+e^{-(0.154613959258218\eta -0.168446269018335)}}\\ +\frac{20.7645374197723}{1+e^{-(1.28504713572369\eta -7.68128721241741)}}+\frac{-6.15181498117780}{1+e^{-(0.640952026512222\eta -14.3337587212102)}}\\ +\frac{10.5776262645944}{1+e^{-(-0.708089052040594\eta +16.0885094681750)}}+\frac{0.491466795443296}{1+e^{-(4.16053391330251\eta +16.2120269404568)}}\\ +\frac{-3.43638618340971}{1+e^{-(-0.943137790572956\eta +0.0553759925406189)}}+\frac{-0.318046406859706}{1+e^{-(6.40549504261589\eta +12.2749020327602)}}, \end{array}$$

(A6) $$\begin{array}{c} g_{c2}\left(\eta \right)=\frac{-8.47317900841952}{1+e^{-(-11.9477047132981\eta -18.1566701360692}}+\frac{-6.33591990117719}{1+e^{-(-0.163084568883509\eta -4.38578114087729)}}\\ +\frac{-0.165779753418781}{1+e^{-(-10.3014537212347\eta -10.0041641527273)}}+\frac{-1.17904389253406}{1+e^{-(-1.38009051089181\eta +1.00333757703818)}}\\ +\frac{8.12169845689494}{1+e^{-(0.825916353174769\eta -2.70915672868049)}}+\frac{-7.37198229475499}{1+e^{-(-0.0234213131749906\eta -13.1476194351527)}}\\ +\frac{15.6703681013559}{1+e^{-(-1.41495646938313\eta -3.09517694217300)}}+\frac{16.3889676382044}{1+e^{-(-1.40304426659244\eta -9.35565379932698)}}\\ +\frac{0.572714326624283}{1+e^{-(1.70245176657341\eta +0.0114206190949522)}}+\frac{-0.594934948505521}{1+e^{-(0.459627953388707\eta -1.23115206064770)}}, \end{array}$$

(A7) $$\begin{array}{c} g_{c3}\left(\eta \right)=\frac{-1.07796758028332}{1+e^{-(-1.28543240703059\eta +0.196053702026635}}+\frac{6.19592319883508}{1+e^{-(-0.143531470530927\eta -10.1054719351599)}}\\ +\frac{1.29028342234795}{1+e^{-(-23.0695603927930\eta -16.0019589180604)}}+\frac{24.5020718437534}{1+e^{-(0.350869473680966\eta -2.26511555868703)}}\\ +\frac{-11.0881470649015}{1+e^{-(0.599588222339071\eta -14.3258787098489)}}+\frac{29.0956646461048}{1+e^{-(6.67545138780301\eta -22.4456035348698)}}\\ +\frac{8.90692480915053}{1+e^{-(1.51624995720002\eta -29.9999999880872)}}+\frac{12.8164183892417}{1+e^{-(-1.65771204510768\eta -2.87334837671866)}}\\ +\frac{1.36781506017920}{1+e^{-(5.20394229806136\eta -24.0355802049677)}}+\frac{-1.69861515711933}{1+e^{-(-3.89342842336576\eta -2.87334837671866)}}, \end{array}$$

(A8) $$\begin{array}{c} g_{c4}\left(\eta \right)=\frac{-1.24713293446349}{1+e^{-(1.53567702451395\eta +3.57669397470700}}+\frac{-0.676680032898537}{1+e^{-(0.655768801828501\eta +17.6555488346129)}}\\ +\frac{-0.390350827947282}{1+e^{-(1.25841119561704\eta +1.11266099970389)}}+\frac{-1.43469475597621}{1+e^{-(1.73771405866776\eta +21.5734062745439)}}\\ +\frac{18.3691722439688}{1+e^{-(-1.66248982379959\eta -3.134135979482099)}}+\frac{0.0368793877160828}{1+e^{-(-10.3410764878639\eta -22.5901794608600)}}\\ +\frac{-0.248119373172110}{1+e^{-(-29.5353912988548\eta -26.0173040624925)}}+\frac{1.17767407311012}{1+e^{-(0.0539736585005905\eta -11.6757725506848)}}\\ +\frac{2.25917320036582}{1+e^{-(1.19164383326462\eta +0.342010952068173)}}+\frac{27.6538708494121}{1+e^{-(0.308494157838889\eta -2.29512336973742)}}. \end{array}$$

Approximated solution of problem 3 for all cases:

(A9) $$\begin{array}{c} g_{c1}\left(\eta \right)=\frac{10.6223016876198}{1+e^{-(-0.0780597051405579\eta -11.0594121935616}}+\frac{3.79819466293440}{1+e^{-(0.861458499363787\eta -0.438470609384736)}}\\ +\frac{0.698741371189724}{1+e^{-(0.786140690777637\eta -8.78130980650411)}}+\frac{11.8499512787238}{1+e^{-(-1.50650777498768\eta -4.61144049518847)}}\\ +\frac{-4.44419822650151}{1+e^{-(0.753724071718736\eta -0.958293015056910)}}+\frac{-9.88990534420985}{1+e^{-(5.83224502578263\eta -29.2024447592141)}}\\ +\frac{0.645221674628451}{1+e^{-(-22.3474380209428\eta -16.1474403196985)}}+\frac{-3.11073670076990}{1+e^{-(1.41635700479857\eta +2.14634670637277)}}\\ +\frac{0.268695863418086}{1+e^{-(0.175996951996260\eta +0.897543573084633)}}+\frac{19.5012559927405}{1+e^{-(0.441361620015144\eta -2.05207094310822)}}, \end{array}$$

(A10) $$\begin{array}{c} g_{c2}\left(\eta \right)=\frac{-6.92890308647009}{1+e^{-(0.0545381880656413\eta +12.2082857209819}}+\frac{-6.23331078235345}{1+e^{-(-0.581378690579424\eta -0.409023904740514)}}\\ +\frac{-29.9324856748492}{1+e^{-(1.78727279077926\eta -11.7743466273260)}}+\frac{12.0136230358825}{1+e^{-(0.708676961683629\eta +4.19290104077455)}}\\ +\frac{1.77880725037356}{1+e^{-(-1.22968746248694\eta -1.83425889073550)}}+\frac{-0.337375691939904}{1+e^{-(-5.40657258361172\eta +19.2451728127842)}}\\ +\frac{-2.85909630821726}{1+e^{-(-5.02180707954942\eta +29.9999981363404)}}+\frac{9.02834428332113}{1+e^{-(-1.34386446057716\eta -3.84329948847535)}}\\ +\frac{8.68032428040273}{1+e^{-(0.651475142466642\eta -3.18673553894291)}}+\frac{-2.69002422724243}{1+e^{-(-12.6647171172223\eta -20.6995737039304)}}, \end{array}$$

(A11) $$\begin{array}{c} g_{c3}\left(\eta \right)=\frac{29.8111938205376}{1+e^{-(-0.0107097290254530\eta -30)}}+\frac{-2.76626499276282}{1+e^{-(-0.587109702702231\eta -1.14253953960096)}}\\ +\frac{-1.33492673261703}{1+e^{-(10.7490644221616,\eta +15.7972328680717)}}+\frac{10.0599018805831}{1+e^{-(0.345153770567496\eta -1.00031317237958)}}\\ +\frac{0.216801087201179}{1+e^{-(-0.00218639525210423,\eta -1.82671560273401)}}+\frac{1.92259401563730}{1+e^{-(1.25701386073571\eta -7.35568198676311)}}\\ +\frac{-0.715596259271990}{1+e^{-(29.9999999949570,\eta +30.0000000545581)}}+\frac{-5.92952657567245}{1+e^{-(0.415410262529314\eta -9.58930991734557)}}\\ +\frac{-2.47384175974838}{1+e^{-(-1.13582523528867,\eta -5.02035265373169)}}+\frac{-10.1321182673046}{1+e^{-(-0.216186234504706\eta -23.3399939748596)}}. \end{array}$$
